# Supplementary material for: An oral cancer vaccine using a Bifidobacterium vector suppresses tumor growth in a syngeneic mouse bladder cancer model
Source: Mol Ther Oncolytics. 2021 Aug 25;22:592–603. doi: 10.1016/j.omto.2021.08.009 (PMC8449024; doi:10.1016/j.omto.2021.08.009)
Supplement: Document 1. Figures S1–S6 [file mmc1.pdf]

OMTO, Volume *22*

Supplemental information

An oral cancer vaccine using a *Bifidobacterium* vector suppresses tumor growth in a syngeneic mouse bladder cancer model

Koichi Kitagawa, Maho Tatsumi, Mako Kato, Shota Komai, Hazuki Doi, Yoshiko Hashii, Takane Katayama, Masato Fujisawa, and Toshiro Shirakawa

Supplemental Information

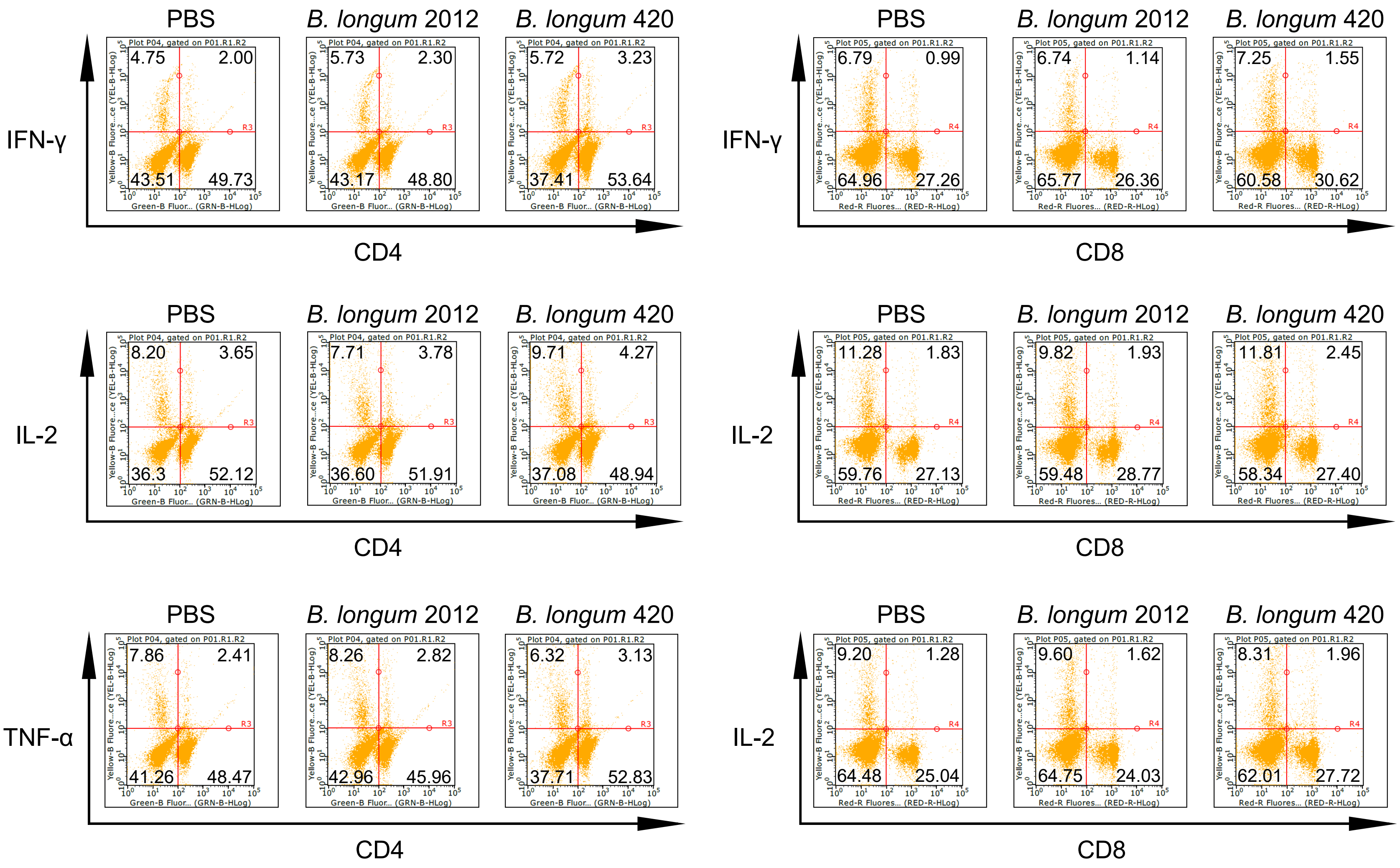

Figure S1. Representative histograms of ICCS for splenocytes

Representative dot plots and gating of intracellular cytokine staining (ICCS) for T cells in splenocytes.

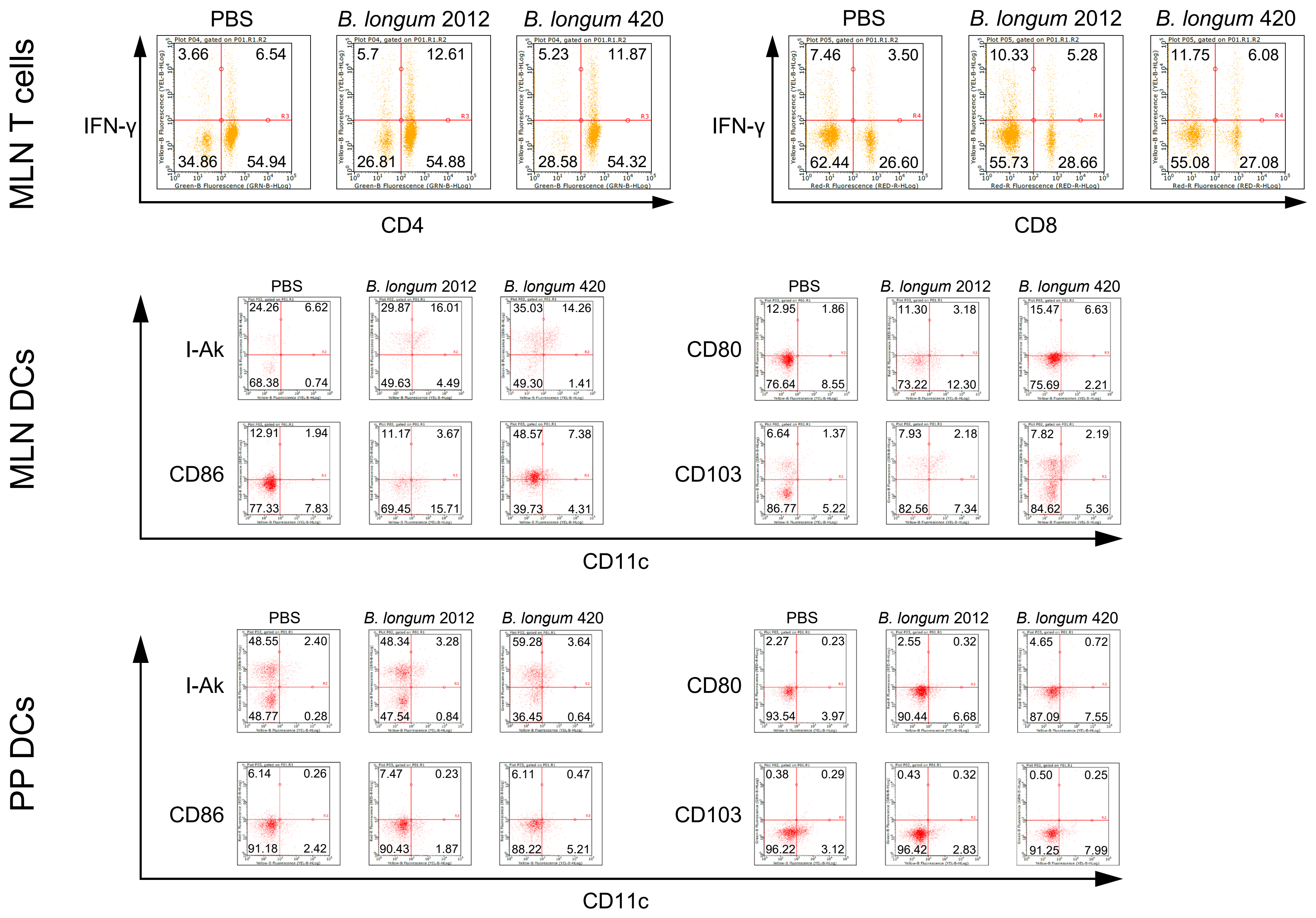

Figure S2. Representative histograms of ICCS for T cells DCs in MLNs and PPs
Representative dot plots and gating for intracellular cytokine staining (ICCS) for T cells in mesenteric lymph nodes (MLNs) and Peyer's patches (PPs), and dendritic cells (DCs) in MLNs and PPs.

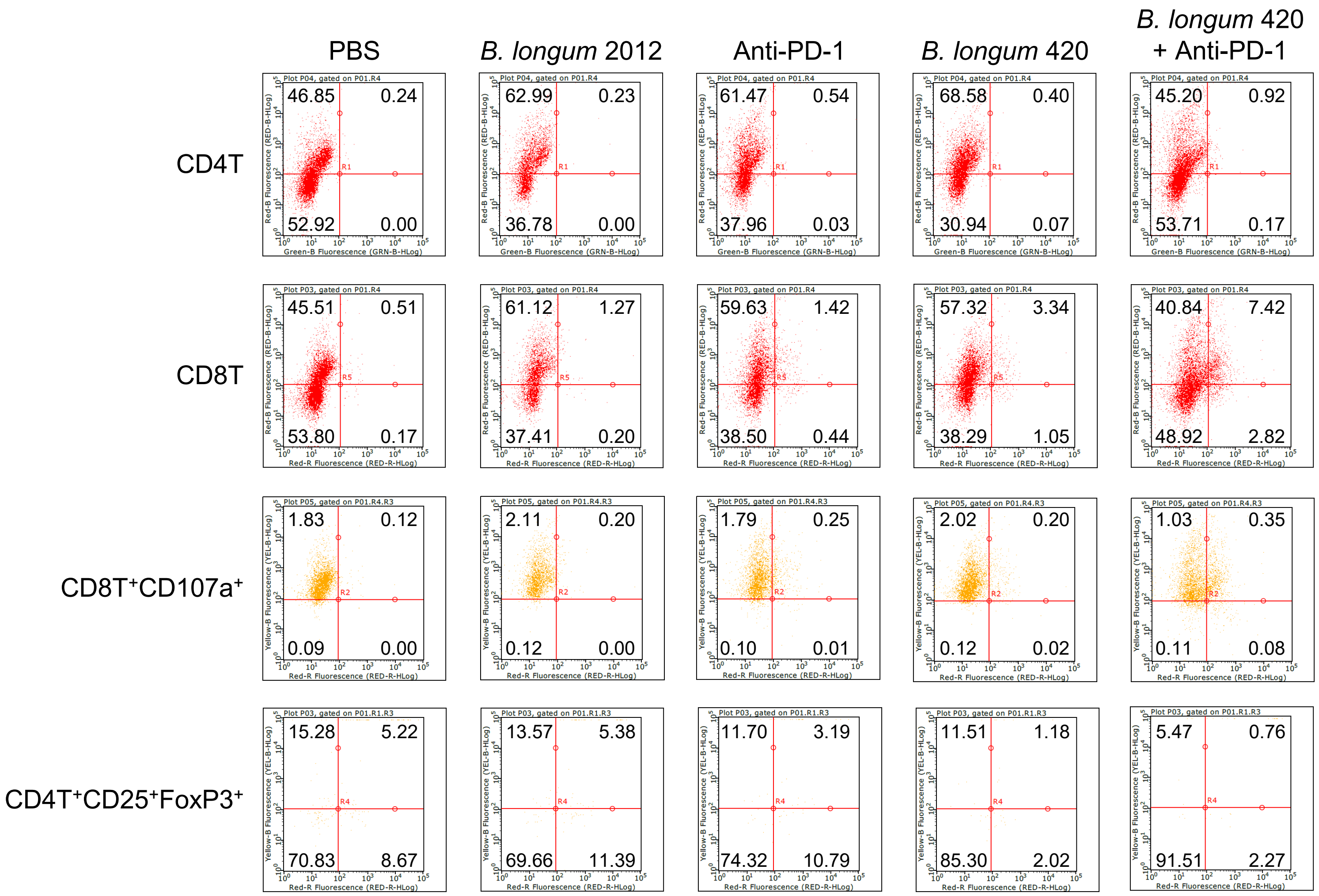

Figure S3. Representative histograms of FMC analysis for TIL in MBT-2 tumor tissues
Representative dot plots and gating of flow cytometric (FCM) analysis for tumor infiltrating lymphocytes (TILs) in MBT-2 tumors after combination therapy with *B. longum* 420 and anti-PD-1 antibody.

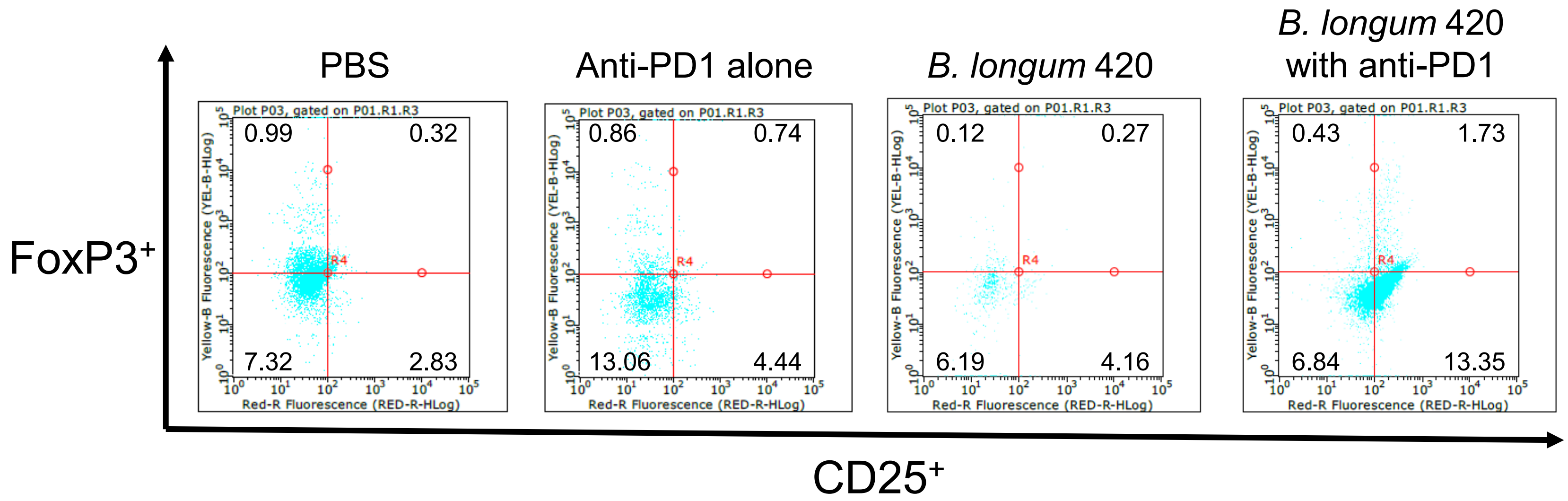

Figure S4. Representative histograms of FMC for TIL in MBT-2 tumor tissues
Representative dot plots and gating of flow cytometric (FCM) analysis for tumor infiltrating regulatory T cells in MBT-2 tumors poorly responsive to anti-PD-1 antibody.

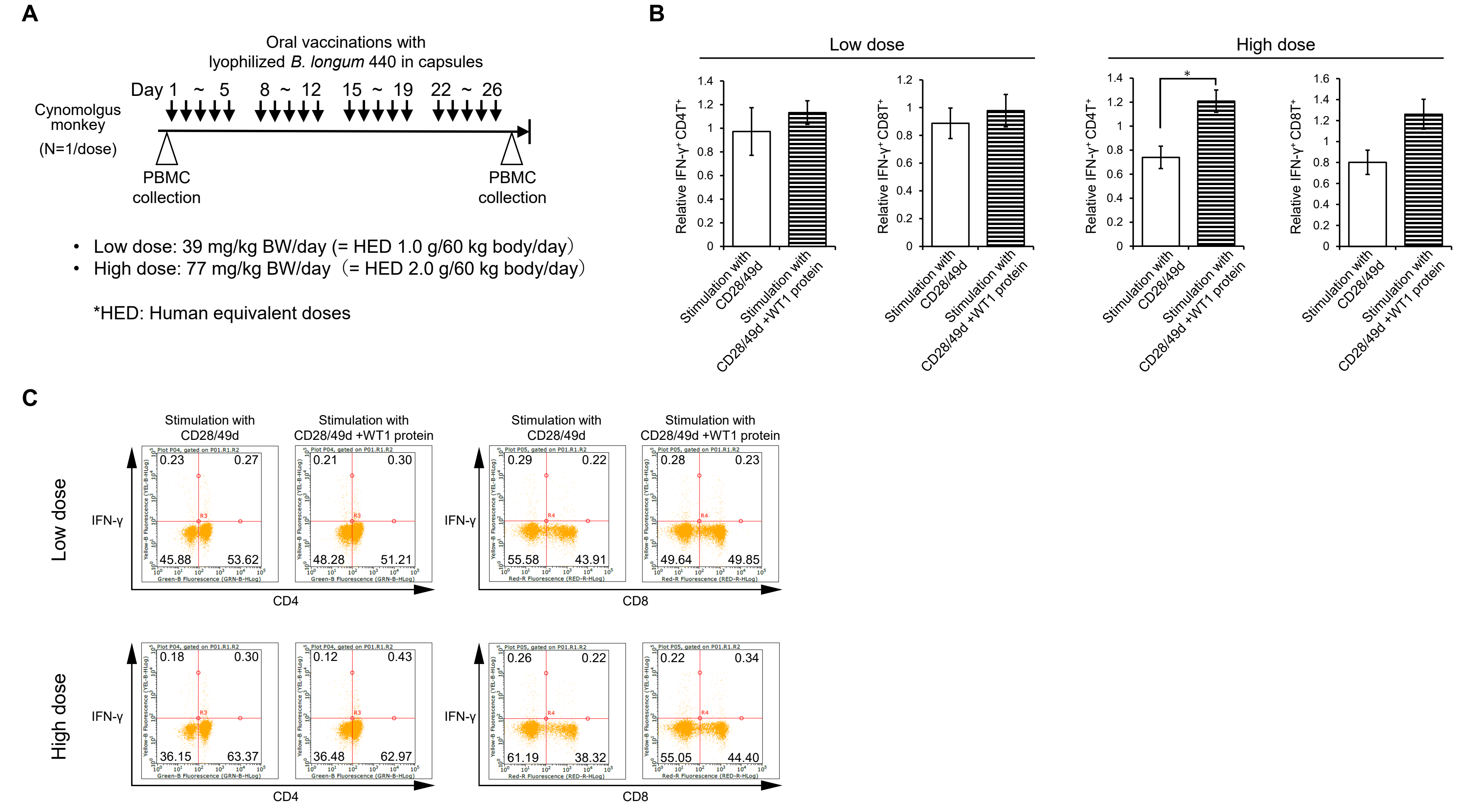

Figure S5. B440 induced WT1-specific T cell response in cynomolgus monkey

The B440, a lyophilized *B. longum* 440 in capsules was orally administrated into cynomolgus monkeys, at a low dose (39 mg/kg/day) and high dose (77 mg/kg/day), which is equivalent to 1.0 g or 2.0 g for human (60 kg/body/day) doses, five times a week for four weeks (A). After final oral administration, peripheral blood mononuclear cells (PBMCs) were isolated for ICCS assay. As a result, the number of CD4$^+$IFN-γ$^+$ T cells was significantly increased after stimulation with WT1 protein in the cynomolgus monkey vaccinated with a high dose of B440 (B). Also, the number of CD8$^+$IFN-γ$^+$ T cells was increased after stimulation with WT1 protein in the cynomolgus monkey vaccinated with high dose B440, but this was not statistically significant (B). (C) Representative histograms and gating for intracellular cytokine staining (ICCS) of monkey PBMCs after oral administration of *B. longum* 440.

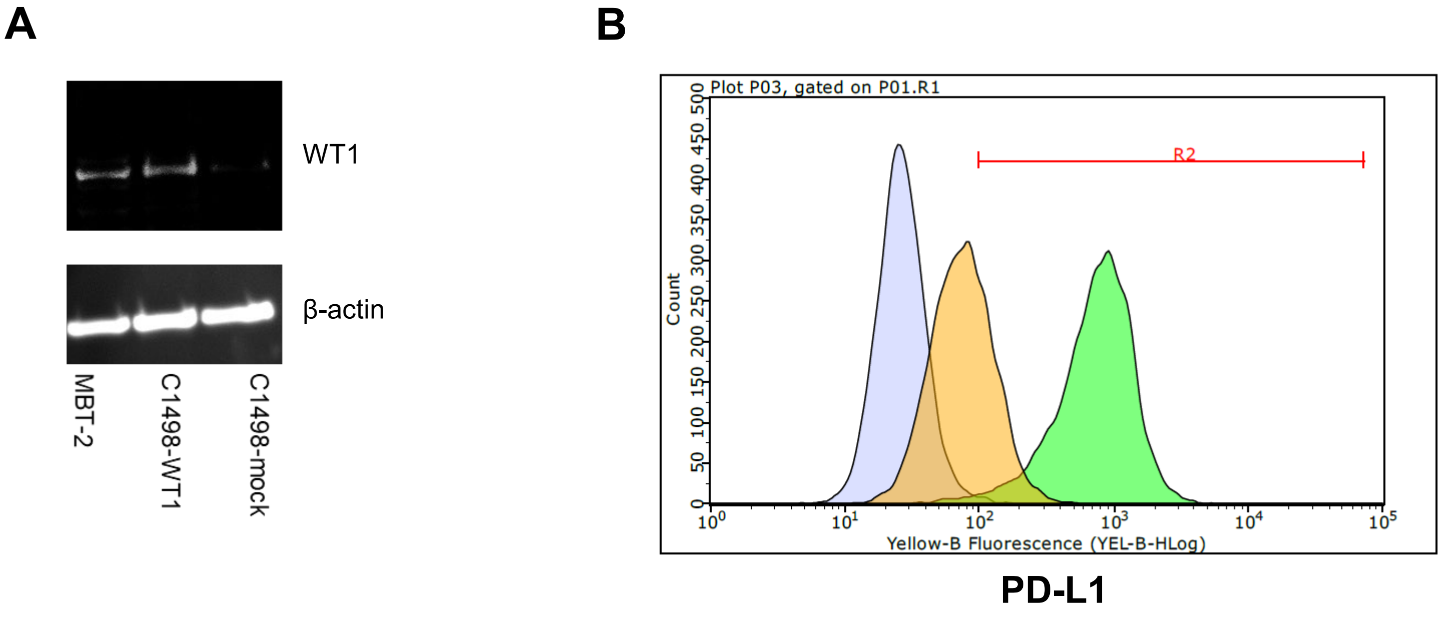

Figure S6. *In vitro* expressions of WT1 and PD-L1 proteins in MBT-2 cells

A) Expression of WT1 protein in MBT-2. The expression of WT1 protein was determined by western blotting. WT1 protein was detected at 55 kDa in MBT-2 and C1498-WT1 but not in C1498-mock.

B) PD-L1 expression on MBT-2 cells. MBT-2 cells were treated with/without IFN-γ and stained with anti-mouse PD-L1 antibody in vitro. Green: MBT-2 cells stained with PD-L1 after culture with 20 ng/ml IFN-γ. Orange: MBT-2 cells stained with PD-L1 after culture with 0 ng/ml IFN-γ Light blue: MBT-2 cells stained with isotype control IgG after culture with 20 ng/ml IFN-γ.
