# Supplementary material for: An oral cancer vaccine using a Bifidobacterium vector suppresses tumor growth in a syngeneic mouse bladder cancer model
Source: Mol Ther Oncolytics. 2021 Aug 25;22:592–603. doi: 10.1016/j.omto.2021.08.009 (PMC8449024; doi:10.1016/j.omto.2021.08.009)
Supplement: Document 2. Article plus supplemental information [file mmc2.pdf]

Molecular Therapy
Oncolytics
Original Article

An oral cancer vaccine using a *Bifidobacterium* vector suppresses tumor growth in a syngeneic mouse bladder cancer model

Koichi Kitagawa,[1] Maho Tatsumi,[1] Mako Kato,[1] Shota Komai,[1] Hazuki Doi,[1] Yoshiko Hashii,[2] Takane Katayama,[3] Masato Fujisawa,[4] and Toshiro Shirakawa[1,4]

[1]Laboratory of Translational Research for Biologics, Department of Advanced Medical Science, Kobe University Graduate School of Science, Technology and Innovation, Kusunoki-cho, Chuo-ku, Kobe 650-0017, Japan; [2]Department of Pediatrics, Osaka University Graduate School of Medicine, Suita 565-0871, Japan; [3]Division of Integrated Life Science, Graduate School of Biostudies, Kyoto University, Kyoto 606-8501, Japan; [4]Department of Urology, Kobe University Graduate School of Medicine, Kobe 650-0017, Japan

Cancer immunotherapy using immune-checkpoint inhibitors (ICIs) such as PD-1/PD-L1 inhibitors has been well established for various types of cancer. Monotherapy with ICIs, however, can achieve a durable response in only a subset of patients. There is a great unmet need for the ICI-resistant-tumors. Since patients who respond to ICIs should have preexisting antitumor T cell response, combining ICIs with cancer vaccines that forcibly induce an antitumor T cell response is a reasonable strategy. However, the preferred administration sequence of the combination of ICIs and cancer vaccines is unknown. In this study, we demonstrated that combining an oral WT1 cancer vaccine using a *Bifidobacterium* vector and following anti-PD-1 antibody treatment eliminated tumor growth in a syngeneic mouse model of bladder cancer. This vaccine induced T cell responses specific to multiple WT1 epitopes through the gut immune system. Moreover, in a tumor model poorly responsive to an initial anti-PD-1 antibody, this vaccine alone significantly inhibited the tumor growth, whereas combination with continuous anti-PD-1 antibody could not inhibit the tumor growth. These results suggest that this oral cancer vaccine alone or as an adjunct to anti-PD-1 antibody could provide a novel treatment option for patients with advanced urothelial cancer including bladder cancer.

INTRODUCTION

The cancer-preventive effects of probiotics, for example, *Lactobacillus* against colorectal cancer, have long been reported.[1] Many novel findings regarding various associations between cancer therapy and gut microbiota have followed recent advances in genetic analysis technology.[2,3] The metagenome analysis of the gut microbiome with next-generation sequencing methods is providing deeper insights into the clinical association between systemic cancer therapy and the gut microbiota.[4] Recent clinical studies revealed the close association between gut microbiota composition and the outcome of immune-checkpoint inhibitors (ICIs) such as anti-PD-1 (program cell death protein 1), anti-PD-L1 (Programmed cell Death 1-Ligand 1), and anti-CTLA-4 (Cytotoxic T-lymphocyte-associated antigen 4) antibodies.[5–7] As a mechanism of action, the regulation of drug metabolism, hematopoiesis, inflammation, and immunity by the gut microbiota influences the efficacy and toxicity of chemotherapy, radiotherapy, and immunotherapy.[8]

One of the scenarios for the immunomodulatory effect of gut microbiota is that systemic cancer therapy causes damage to the intestinal mucosa and translocation of the intestinal bacteria to the gut-associated lymphoid tissues (GALTs), which enhances immune responses. As a combination of cancer therapy and intestinal bacteria in that scenario, cisplatin or oxaliplatin and *Lactobacillus acidophilus*,[9] cyclophosphamide and *Enterococcus hirae*,[10] total body irradiation (TBI) and Gram-negative bacteria,[11] and anti-CTLA-4 antibody and *Bacteroides fragilis*[12] have been previously reported. In addition, some bacterial species such as *Salmonella enterica*, which has a type three secretion system (TTSS) to penetrate mucosal membranes, can translocate to GALT without cancer therapy. Utilizing this natural tropism, *Salmonella enterica* has been used as a bacterial vector for an oral vaccine platform to deliver a heterologous antigen to the gut immune system.[13–15] Besides *Salmonella*, lactic acid bacteria (LAB), which have a strong mucosal adhesive ability, including *Lactobacillus*, have been used as bacterial vectors for an oral vaccine platform.[16]

We also developed an oral vaccine platform using LAB, *Bifidobacterium longum* (*B. longum*), and constructed a variety of oral vaccines, including a recombinant *B. longum* displaying *Salmonella* flagellin protein as a typhoid vaccine,[17] a recombinant *B. longum* displaying hepatitis C virus (HCV) nonstructural protein 3 (NS3) as an HCV

Received 26 May 2021; accepted 19 August 2021;
https://doi.org/10.1016/j.omto.2021.08.009.

Correspondence: Toshiro Shirakawa, MD, PhD, Laboratory of Translational Research for Biologics, Department of Advanced Medical Science, Kobe University Graduate School of Science, Technology and Innovation, Kusunoki-cho, Chuo-ku, Kobe 650-0017, Japan.
E-mail: toshiro@med.kobe-u.ac.jp

 © 2021 The Author(s).
This is an open access article under the CC BY license (http://creativecommons.org/licenses/by/4.0/).

vaccine,[18] and a recombinant *B. longum* displaying Wilms' tumor 1 (WT1) protein as a cancer vaccine.[19] The WT1 gene encodes a zinc finger transcription factor, plays an important role for the normal development of urogenital organs, and is overexpressed in various tumors, such as leukemia, colon cancer, breast cancer, prostate cancer, urothelial cancer, and pediatric kidney tumors (Wilms' tumor).[20,21] In addition, WT1 was ranked as the No. 1 antigen among 75 tumor antigens by the National Cancer Institute pilot project developing a priority list of tumor vaccine target tumor-associated antigens.[22]

Reportedly, after the oral administration of *B. pseudocatenulatum* (bp) to mice, bp cells were detected with dendritic cells (DCs) in Peyer's patch (PP) and mesenteric lymph nodes (MLNs) within 1 and 20 h, respectively.[23] Therefore, *Bifidobacterium* is a suitable bacterial vector to deliver heterologous antigen to the gut mucosal immune system. Furthermore, a positive association between *B. longum* and anti-PD-1 efficacy in metastatic melanoma patients was found in a clinical study.[24] The *B. longum* platform may have an adjuvant effect alongside ICI cancer immunotherapy.

In our previous studies, we confirmed that recombinant *B. longum* displaying a mouse WT1 protein inhibited the tumor growth of mouse syngeneic prostate cancer cells *in vivo* and that its antitumor activity could be augmented by an anti-PD-1 antibody.[25] In this study, the combination of this oral vaccine and an anti-PD-1 antibody that follows the oral vaccine demonstrated the complete regression of mouse syngeneic MBT-2 bladder cancer cell tumors. Reportedly, MBT-2 cells naturally express WT1 protein.[26] To address the great unmet need for a treatment for anti-PD-1 antibody-resistant or -poorly responsive tumors, and to identify the preferred administration sequence of this combination therapy, we performed an experiment using a tumor model poorly responsive to an initial anti-PD-1 treatment. In that experimental model, we found that an oral vaccine alone significantly inhibited the tumor growth, whereas a combination of the oral vaccine and continuous anti-PD-1 antibody treatment could not inhibit tumor growth after the initial treatment with anti-PD-1 antibody. Interestingly, a significantly higher number of regulatory T (Treg) cells was detected in the tumor tissues of combination treatment mice compared to the oral vaccine alone treatment mice.

Although ICIs are being used as a standard of care for various types of advanced cancer, the response rates are mostly limited to 20%–30% when used as a monotherapy.[27,28] Indeed, the objective response rate (ORR) for pembrolizumab (anti-PD-1 antibody) monotherapy in advanced urothelial cancer, including bladder cancer, was reported to be 21.1% in a phase III clinical trial.[29] This WT1 oral cancer vaccine alone or as an adjunct to anti-PD-1 antibody could provide a novel treatment option for patients with advanced urothelial cancer.

RESULTS

B. longum 420 induced tumor-specific cellular immunity through the gut immune system

To determine whether oral administration of *B. longum* 420 (a recombinant *Bifidobacterium longum* displaying a mouse WT1 partial protein) could induce MBT-2 cell-specific cellular immunity through the gut immune system, we orally administrated *B. longum* 420 to C3H/He mice and isolated splenocytes or lymphocytes from spleen, MLN, and PP to perform immunological assays. After administration of *B. longum* 420, *B. longum* 2012 (a recombinant *Bifidobacterium longum* displaying galacto-*N*-biose/lacto-*N*-biose I binding protein [GL-BP] anchor protein without a WT1 protein), or phosphate-buffered saline (PBS), splenocytes and lymphocytes were collected, cultured, and stimulated with mitomycin-C-treated MBT-2 cells, a mouse bladder cancer cell line, or Db126 or Mp235 peptides,[30] which are known WT1-specific CD8 epitopes for both human and mouse.

In result, the cell proliferation rate (stimulation index [SI]: cell numbers with stimulation/cell numbers without stimulation) of the splenocytes was significantly higher in the *B. longum* 420 group than the other groups when cells were stimulated with MBT-2 and Mp235 peptide ($p < 0.01$, Figure 1A).

Also, the splenocytes isolated from mice vaccinated with *B. longum* 420 induced a significantly higher increase of interferon-γ (IFN-γ) secretion by stimulation with mitomycin-C-treated MBT-2 compared to the other groups by enzyme-linked immunosorbent assay (ELISA) ($p < 0.05$, Figure 1B). These results indicated that *B. longum* 420 can activate the splenocytes along with IFN-γ secretion, which plays an important role in Th1 immune responses,[31] by stimulation with MBT-2 cells.

In an *in vitro* cytotoxicity assay using splenocytes as effector cells, *B. longum* 420 showed significantly higher CTL activities against MBT-2 target cells compared to PBS at effector-to-target ratios of 20:1, 10:1, and 5:1 ($p < 0.05$, Figure 1C) and significantly higher cytotoxicity compared to *B. longum* 2012 at the effector-to-target cell ratio of 5:1 (Figure 1C, $p < 0.05$).

In addition, to measure the frequency of cytokine-expressing T cells induced by vaccination with *B. longum* 420, we performed intracellular cytokine staining (ICCS) using splenocytes stimulated with mitomycin-C-treated MBT-2. The frequency of $CD4^+$ and $CD8^+$ T cells expressing IFN-γ in the *B. longum* 420-treated mouse group was significantly higher than in the other groups (Figure 1D, $p < 0.05$). Also, the frequency of tumor necrosis factor alpha (TNF-α)-producing $CD8^+$ T cells in the *B. longum* 420-treated mouse group was significantly higher than the other groups.

We next performed an ICCS assay for lymphocytes isolated from MLNs and PPs to investigate the immune responses in GALT. The frequency of $CD4^+$ T cells expressing IFN-γ in MLN was significantly increased in the *B. longum* 420 and *B. longum* 2012 groups compared to the PBS group ($p < 0.01$, Figure 1E), and the frequency of $CD8^+$ T cells expressing IFN-γ in MLN was significantly increased in the *B. longum* 420 group compared to the PBS group ($p < 0.05$, Figure 1E). Regarding activation of DCs in GALT, we determined the frequency of $CD11c^+$ DCs co-expressing I-Ak (major histocompatibility complex [MHC] class II molecule of C3H/He mice), CD80, CD86, or

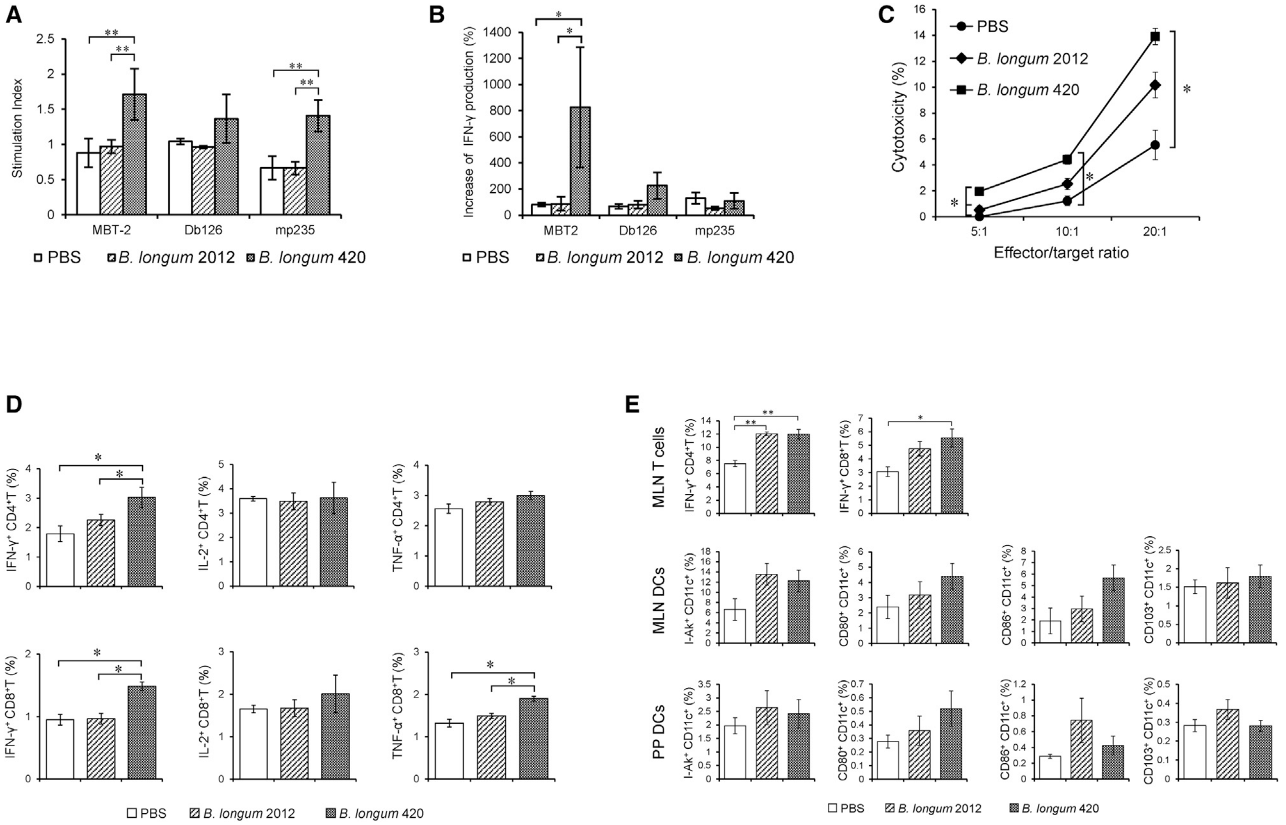

Figure 1. Analyses of splenocytes and lymphocytes isolated from vaccinated mice

(A) Splenocyte proliferation after oral vaccination with *B. longum* 420. After completion of the vaccination, splenocytes in all three groups (*B. longum* 420, *B. longum* 2012, and PBS; n = 5) were cultured and stimulated with mitomycin C-treated MBT-2, Db126 peptide (RMFPNAPYL), or Mp235 peptide (CMTWNQMNL) for 48 h *in vitro*. Cell proliferation was determined by XTT assay (*p < 0.05). Each data point represents the average of the stimulation index (SI), which is the ratio of stimulated cell number divided by non-stimulated cell number (bars, ±SE). (B) Cytokine secretion in splenocytes from mice vaccinated with *B. longum* 420. After completion of the vaccination, splenocytes in all three groups (*B. longum* 420, *B. longum* 2012, and PBS; n = 5) were cultured and stimulated with mitomycin C-treated MBT-2, Db126 peptide (RMFPNAPYL), or Mp235 peptide (CMTWNQMNL) for 48 h *in vitro*. IFN-γ secretion was determined by ELISA (*p < 0.05). Each data point represents the average of the relative concentrations of IFN-γ: the concentration of stimulated cells divided by that of non-stimulated cells (bars, ±SE). (C) *In vitro* CTL assay. After completion of the vaccination, splenocytes in all three groups (*B. longum* 420, *B. longum* 2012, and PBS; n = 5) were cultured and stimulated with IL-2- and mitomycin C-treated MBT-2 cells for 6 days *in vitro*. After the stimulation, the splenocytes were co-cultured with target MBT-2 cells for 8 h at ratios of 5:1, 10:1, and 20:1. The cytotoxicity of effector cells (the splenocytes) against target cells (MBT-2 cells) is shown (*p < 0.05). Each data point represents the average of % cell lysis (cytotoxicity; bars, ±SE). (D) Intracellular cytokine staining (ICCS) for measuring the cell numbers of cytokine-expressing $CD4^+$ and $CD8^+$ T cells. After completion of the vaccination, splenocytes in all three groups (*B. longum* 420, *B. longum* 2012, and PBS; n = 5) were cultured and stimulated with mitomycin C-treated-MBT-2 cells for 38 h *in vitro*. The frequencies of IFN-γ-producing, IL-2-producing, or TNF-producing $CD4^+$ and $CD8^+$ T cells were measured by ICCS (*p < 0.05). Each data point represents the average of the cell frequencies (bars, ±SE). (E) ICCS for measuring T cells and DCs in MLNs and PPs after oral vaccination. After 2 weeks or 4 weeks of completion of the vaccination, splenocytes in all three vaccination groups (*B. longum* 420, *B. longum* 2012, and PBS; n = 5) were cultured and stimulated with mitomycin C-treated MBT-2 cells for 6 h *in vitro*. The frequencies of IFN-γ-expressing $CD4^+$ and $CD8^+$ T cells were determined by ICCS (*p < 0.05). Each data point represents the average of the cell frequencies (bars, ±SE). DCs were also isolated from MLNs and PPs and stained for I-Ak, CD103, CD11c, CD80, or CD86. The frequencies of I-Ak^+, $CD103^+$, $CD80^+$ or $CD86^+$ on $CD11c^+$ DCs were detected (*p < 0.05). Each data point represents the average of the cell frequencies (bars, ±SE).

CD103 in MLNs and PPs. Although none of the increases of $CD11c^+$ cells was significant, *B. longum* 420 and *B. longum* 2012 tended to increase the activated DCs in MLNs and PPs (Figure 1E). These ICCS data indicated that *B. longum* 420 could induce activated $CD4^+$ and $CD8^+$ T cells in spleen and MLNs and might activate the DCs in MLNs and PPs. Furthermore, *B. longum* 2012, which does not express a heterologous antigen, could induce activated $CD4^+$ T cells in MLN and might activate the DCs in MLNs and PPs. It has been reported that *Bifidobacterium* can modulate the gut immune system.[32] Representative histograms of each treatment group and gating for ICCS are shown in Figures S1 and S2.

B. longum 420 induced multiple WT1-epitopes-specific immune responses

To identify the MHC class I and class II epitopes in the WT1 protein displayed in *B. longum* 420, we performed epitope peptide screening

A

WT1 amino acid sequence in *B. longum* 420

PSQASSGQA**RMFPNAPYL**PSCLESQPTIRNQGYSTVTFDGAPSYGHTPSHHAAQFPNHSF**KHEDPMG**
QQGSLGEQQYSVPPPVYGCHTPTDSCTGSQALLLRT**PYSSDNLYQMTSQLE****CMTWNQMNL**GATLKG
MAAGSSSSVKW**TEGQSNHGI**GYE**SENHTAPI**LCGAQYRIHTHGVFRG**IQDVRRVSGV****APTL**VRSASET
SEKRPFMCAYPGCNKRYFKLSHLQMHSRKHTGEKP**YQCDFKDCERRFSRS**DQLKRHQRRHTGVKPF
QCKTCQRKFSRSDHLKTHTRTHTGKTSEKPFSCRWHSCQKKFARS**DELVRHHNM**HQ

Red: MHC class I epitopes, Green: MHC class II epitopes

B

WT1 epitopes used in this study

Aa in WT1 protein	Aa in WT1 in *B. longum* 420	Sequences	Score	MHC haplotypes
126-134	10-18	RMFPNAPYL	2	H-2Db, HLA-A*02:01
177-191	61-75	KHEDPMGQQGSLGEQ	18	I-A^k
220-234	104-118	PYSSDNLYQMTSQLE	22	I-A^k
235-243	119-127	CMTWNQMNL	1	HLA-A*24:02
261-269	145-153	TEGQSNHGI	23	H-2K^k
273-280	157-164	SENHTAPI	22	H-2K^k
296-310	180-194	GIQDVRRVSGVAPTL	18	I-A^k
298-306	182-190	QDVRRVSGV	18	H-2K^k
332-347	216-231	KRYFKLSHLQMHSRKH	0	HLA-DRB1*0405
353-367	237-251	YQCDFKDCERRFSRS	18	I-A^k
429-437	313-321	DELVRHHNM	16	H-2K^k

C

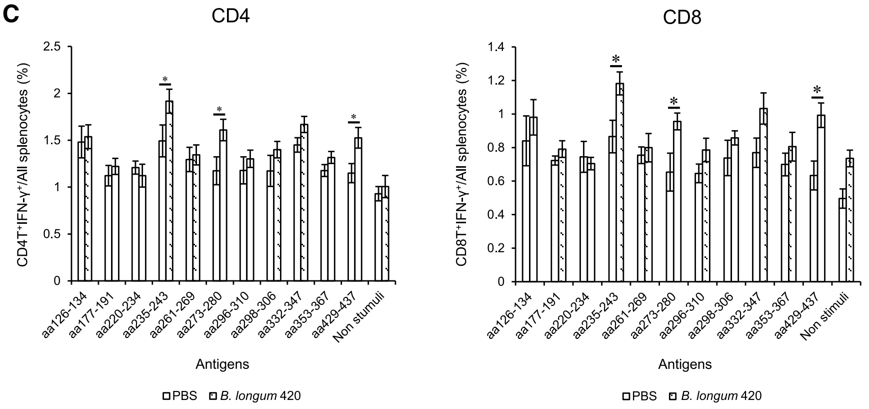

Figure 2. Epitope peptide screening by ICCS

(A) WT1 amino acid sequence in *B. longum* 420. Partial length mouse WT1 protein was transduced into the *B. longum* 105A strain to construct *B. longum* 420-expressing murine WT1. (B) WT1 epitopes used to stimulate the splenocytes *in vitro* after vaccination. (C) ICCS for the WT1-epitope-specific T cells. After completion of the vaccination, splenocytes in the two treatment groups (*B. longum* 420 and PBS; n = 5) were cultured and stimulated with the WT1-epitope peptides shown in (B) for 38 h *in vitro*. The frequencies of IFN-γ-expressing-CD4$^+$ and CD8$^+$ T cells were determined by ICCS (*p < 0.05). Each data point represents the average of the cell frequencies (bars, ±SE).

by ICCS. The *B. longum* 420 vaccine displays most of the mouse WT1 protein (aa117–419, Figure 2A) and contains several known MHC class I and class II epitopes of WT1 protein including Db126 (aa126–134, RMFPNAPYL) as a MHC class I epitope in both human (HLA-A*0201) and mouse (C57BL/6; H-2Db), mp235 (aa235–243, CMTWNQMNL) as a MHC class I epitope in humans (HLA-A*24:02),[30] and WT1-332 (aa332–347, KRYFKLSHLQMHSRKH) as a MHC class II binding epitope in humans (HLA-DRB1*0405).[33] In addition to these three WT1 epitope peptides, we synthesized eight WT1 epitope peptides binding to H-2K^k (MHC class I of C3H/He mice) or I-Ak (MHC class II of C3H/He mice), which were predicted as the top four high-scoring peptides each for H-2K^k and I-Ak by the SYFPEITHI epitope prediction system (http://www.syfpeithi.de/0-Home.htm) (Figure 2B). For epitope screening, splenocytes were isolated from mice vaccinated with *B. longum* 420 or PBS and cultured and stimulated with one each of the 11 epitope peptides (Figure 2B). The IFN-γ-expressing CD4$^+$ and CD8$^+$ T cells in the stimulated splenocytes were measured by ICCS assay. The frequency of CD8$^+$ and CD4$^+$ T cells expressing IFN-γ in the *B. longum* 420-treated mouse group was significantly higher than in the PBS group when splenocytes were stimulated with epitopes of aa235–243 (mp235), aa273–280, and aa429–437 ($p < 0.05$, Figure 2C). These results indicate that *B. longum* 420 can induce multiple (at least three) WT1 epitopes specific for CD4$^+$ and CD8$^+$ T cells in C3H/H3 mice.

B. longum 420 followed by anti-PD-1 antibody completely suppressed MBT-2 tumor growth

To investigate the synergistic antitumor activity of a combination of *B. longum* 420 and following anti-PD-1 antibody, we conducted animal studies using the MBT-2-bearing C3H/He mouse bladder cancer syngeneic tumor model. Thirty-five mice subcutaneously inoculated with MBT-2 cells were randomly assigned to 5 treatment groups (n = 7): *B. longum* 420+anti-PD-1 antibody, *B. longum* 420 alone, *B. longum* 2012 alone, PBS+anti-PD-1 antibody, and PBS at 7 days after the tumor inoculation. We orally administered 1.0×10^9 colony-forming units (CFU) of *B. longum* 420 or *B. longum* 2012 five times a week for 5 weeks (days 7–11, 14–18, 21–25, 28–32, and 35–39) and intraperitoneally injected 200 μg of anti-mouse PD-1 antibody five times in total on days 11, 14, 17, 21, and 24 (Figure 3A). Oral administration of *B. longum* 420 combined with following intraperitoneal injection of mouse anti-PD-1 antibody completely suppressed the growth of MBT-2 tumors and cured all seven mice, whereas *B. longum* 420 alone cured three out of seven mice (Figure 3B). In the other groups (n = 7), all mice died from tumor growth, with an average survival of 28.4 ± 0.7 (standard error) days with PBS control, 30.3 ± 2.5 days with *B. longum* 2012, and 36.0 ± 4.4 days with anti-PD-1 antibody alone (Figure 3B). The complete responses (CRs) of all seven mice in the combination group and three out of seven mice in the *B. longum* 420-alone group were confirmed until day 117, when observation was discontinued. The survival curve (Figure 3C) for the combination

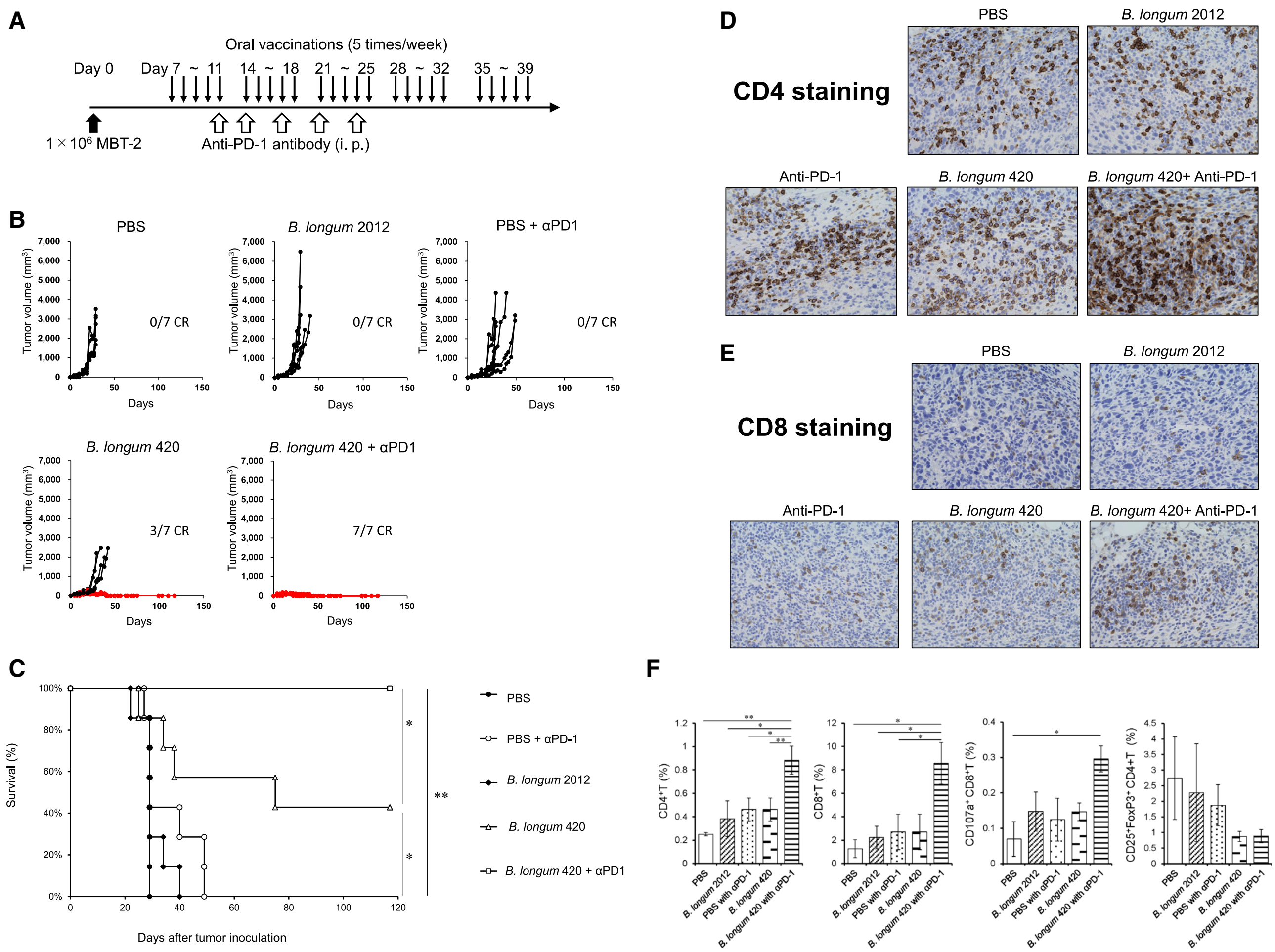

Figure 3. Combination treatment in a syngeneic mouse bladder cancer model

(A) Treatment schedule for oral administration of *B. longum* 420 with anti-PD-1 antibody. Mice (n = 7) were orally vaccinated with *B. longum* 420, *B. longum* 2012, or PBS 5 times a week for 4 weeks after the tumor inoculation with MBT-2 cells. Anti-PD-1 antibody or IgG isotype control was intraperitoneally injected into mice at days 11, 14, 17, 21, and 24 after tumor inoculation. (B). *In vivo* antitumor effects of oral administration of *B. longum* 420 and intraperitoneal injection of anti-PD-1 antibody. Tumor growth curves of MBT-2 of individual mice from the following treatment groups are presented: PBS, PBS+anti-PD-1 antibody, *B. longum* 2012, *B. longum* 420, *B. longum* 420+anti-PD-1, and *B. longum* 420+IgG isotype control. The growth curves of mice that had complete responses (CRs) are shown in red. (C) Kaplan-Meyer survival curve. The combination treatment of *B. longum* 420 and anti-PD-1 significantly improved survival compared to the other treatment groups (*$p < 0.05$, **$p < 0.01$). *B. longum* 420 alone significantly improved survival compared to PBS, PBS+anti-PD-1 antibody, and *B. longum* 2012 groups (*$p < 0.05$). (D and E) Immunohistochemical staining for tumor-infiltrating T cells in MBT-2 tumors. Resected MBT-2 tumors were immunohistochemically stained with anti-CD4 antibody or anti-CD8 antibody. Representative pictures of immunohistochemical staining in each treatment group are shown (400×). (F) Tumor-infiltrating T cells in MBT-2 tumor. After treatment, tumors were resected and stained with CD3, CD4, CD8, CD25, CD107a, and FoxP3. The proportion of tumor-infiltrating T cells was determined by flow cytometry. Each data point represents the average of the cell frequencies (*$p < 0.05$, **$p < 0.01$, bars, ±SE).

of *B. longum* 420 with following anti PD-1 antibody treatment showed a significant prolongation of survival compared with the other treatment groups ($p < 0.05$ or $p < 0.01$, Figure 3C). Also, *B. longum* 420 alone significantly prolonged the survival period compared to the *B. longum* 2012 and PBS groups ($p < 0.05$, Figure 2C).

We next collected tumor tissues from another set of mouse treatment groups after treatment to investigate the tumor infiltrating lymphocytes (TILs). Immunohistochemical staining for $CD4^+$ and $CD8^+$ T cells showed remarkably increased numbers of $CD4^+$ (Figure 3D) and $CD8^+$ (Figure 3E) T cells infiltrating into MBT-2 tumor tissues in mice treated with the combination of *B. longum* 420 and following anti-PD-1 antibody compared to the other treatment groups. Furthermore, the flow cytometric analysis of T cells isolated from tumor tissues revealed that the combination of *B. longum* 420 and following anti-PD-1 antibody treatment significantly increased tumor-infiltrating $CD4^+$ and $CD8^+$ T cells compared to the other treatment groups ($p < 0.05$, Figure 3F). In addition, the number of tumor-infiltrating $CD107a^+CD8^+$ T cells, which is a marker for

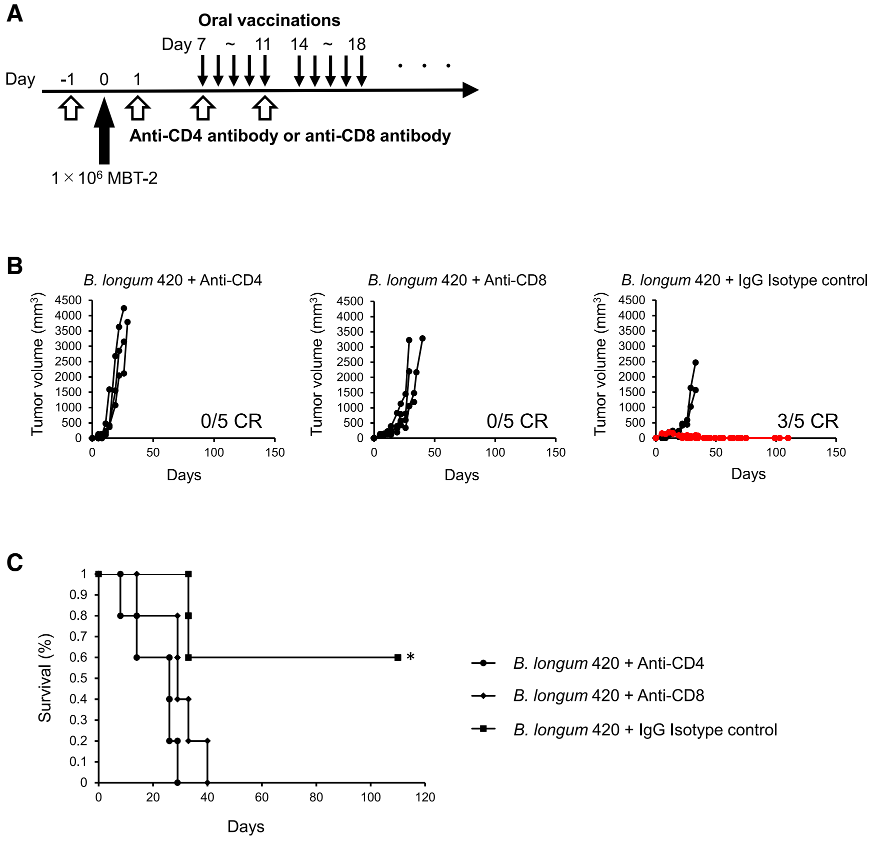

Figure 4. CD4 and CD8 T cells depletion study

(A) Treatment schedule for CD4 and CD8 depletion in the MBT-2 model. Mice were intraperitoneally injected with anti-CD4, anti-CD8, or IgG isotype control at days −1, 1, 7, and 11 (n = 5, respectively). At day 0, 1×10^6 MBT-2 were injected into mice, and recombinant *B. longum* 420 was orally administrated 5 times a week over the following weeks. (B) Depletion of $CD4^+$ and $CD8^+$ T cells *in vivo* in the MBT-2 model. $CD4^+$ and $CD8^+$ T cells were depleted by intraperitoneal injection of anti-CD4 antibody and anti-CD8 antibody, respectively. Tumor growth curves of MBT-2 in an individual mouse from the *B. longum* 420+anti-CD4, *B. longum* 420+anti-CD8, and *B. longum* 420+IgG isotype control treatment groups are shown. The growth curves of mice that had complete responses (CRs) are shown in red. (C) Kaplan-Meyer survival curve. *B. longum* 420 alone significantly prolonged the survival compared to *B. longum* 420 with anti-CD4 antibody or anti CD8 antibody (*p < 0.05).

degranulation of natural killer (NK) and activated $CD8^+$ T cells,[34] was significantly increased compared to the PBS control group ($p < 0.05$, Figure 3F). Interestingly, $CD4^+$ $CD25^+FoxP3^+$ Treg cells were decreased in tumor tissues from the *B. longum* 420 and anti-PD-1 antibody combination and *B. longum* 420-alone groups compared to the other groups (Figure 3F). Representative histograms of ICCS in each treatment group are presented in Figure S3.

$CD4^+$ and $CD8^+$ T cells play a critical role in the antitumor activity of *B. longum* 420

We observed that *B. longum* 420 combined with anti-PD-1 antibody significantly increased the numbers of tumor-infiltrating $CD4^+$ and $CD8^+$ T cells *in vivo*. To confirm the role of $CD4^+$ and $CD8^+$ T cells in the antitumor activity of *B. longum* 420, we next performed a $CD4^+$ and $CD8^+$ T cell depletion study. Intraperitoneal administration of anti-CD4 or anti-CD8 depletion antibodies or IgG isotype control antibody was started a day before MBT-2 tumor inoculation, and oral administration of *B. longum* 420 was started 7 days after the tumor inoculation (Figure 4A). Three out of five mice showed complete regression of tumor in the *B. longum* 420 and IgG isotype control group, whereas all mice died from tumor growth in the *B. longum* 420 and anti-CD4 or anti-CD8 depletion antibody groups (Figure 4B). The survival curve for mice treated with *B. longum* 420 and Ig G isotype control showed significantly prolonged survival compared to the mice treated with *B. longum* 420 and anti-CD4 or anti-CD8 depletion antibodies ($p < 0.05$, Figure 4C). These results strongly indicated that both $CD4^+$ and $CD8^+$ T cells play a critical role in the antitumor activity of *B. longum* 420.

B. longum alone significantly inhibited the growth of tumor poorly responsive to anti-PD-1 antibody

There is a great unmet need for clinical treatments for anti-PD-1 antibody-resistant or -poorly responsive tumors, and it is important to identify the preferred administration sequence of this combination therapy. Therefore, we carried out an experiment using a tumor model poorly responsive to an initial anti-PD-1 antibody treatment. After the initial treatment with anti-PD-1 antibody, a total of 20 out of 60 mice were selected as mice harboring anti-PD-1 antibody-poorly responsive tumors by tumor volume (larger than 450 mm^3) for further evaluation, and the other mice harboring small tumor volume (less than 450 mm^3, as anti-PD-1 antibody responsive) or too large volume (not applicable for evaluation) to be evaluated were excluded (Figures 5A and 5B). The selected 20 mice were treated with PBS, anti-PD-1 antibody alone, *B. longum 420* alone, or a combination of anti-PD-1 antibody and *B. longum* 420 (n = 5, Figure 5B). In result, *B. longum* 420 alone significantly inhibited tumor growth compared to the PBS control group, whereas the combination of anti-PD-1 antibody and *B. longum* 420 or anti-PD-1 antibody alone did not show a significant tumor growth inhibitory effect compared to the PBS group (Figure 5C). Interestingly, in the TIL analysis, the number of tumor-infiltrating Treg ($CD25^+FoxP3^+$) cells in the combination group was significantly higher than in the *B. longum* 420 alone and PBS groups. However, there was no significant difference in the cell frequencies of $CD4^+$ and $CD8^+$ T cells among all treatment groups (Figure 5D). These results suggest that the continuous administration of anti-PD-1 antibody might activate Treg cells, and *B. longum* 420 without anti-PD-1 antibody treatment could have antitumor activity superior

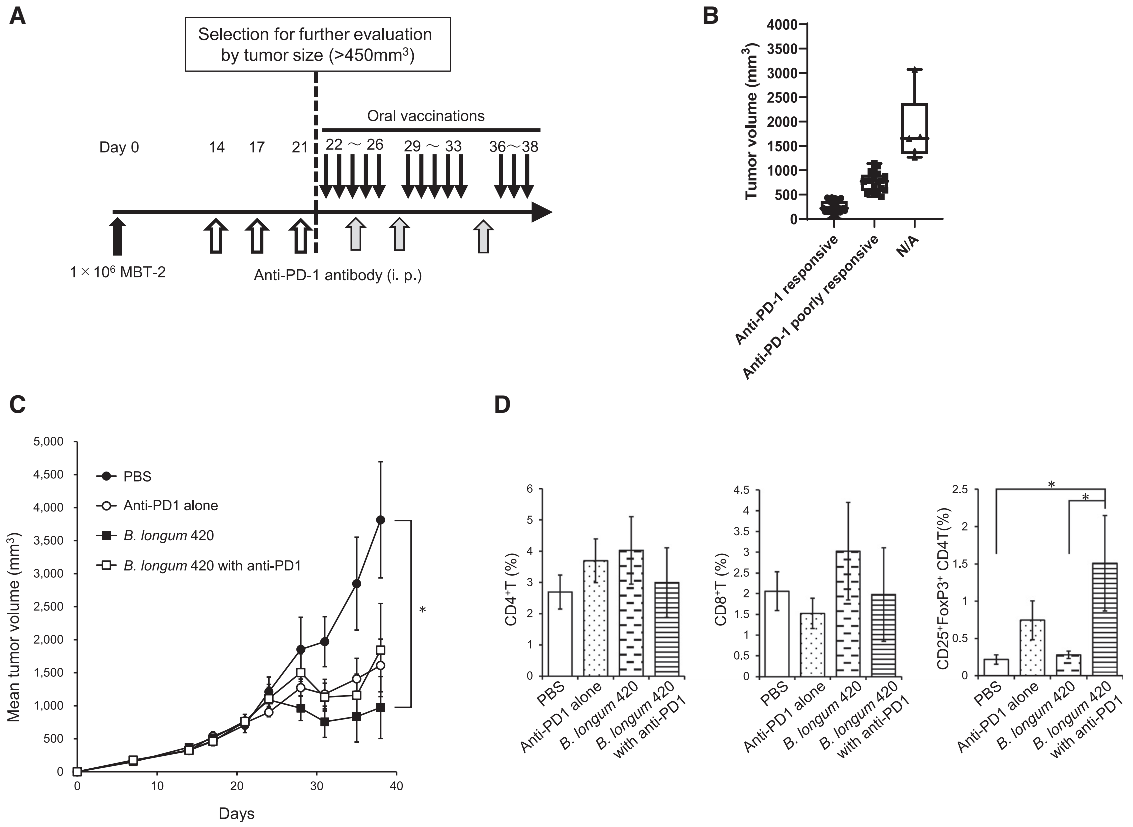

Figure 5. Combination treatment in an anti-PD-1 antibody-poorly responsive tumor model

(A) Treatment schedule for the PD-1-resistant MBT-2 model. At day 0, 1 × 10^6 MBT-2 cells were injected into mice, and then anti-PD-1 antibody was intraperitoneally injected into mice at days 14, 17, and 21 to acquire PD-1 resistance. From day 22, oral vaccination of recombinant *B. longum* 420 was carried out for the following weeks with or without anti-PD-1 therapy (n = 5). (B) A dot plot graph of the individual tumor volumes of the 60 mice at day 20. Anti-PD-1 antibody-responsive tumors were defined as tumor volumes smaller than 450 mm^3 in 35 mice and anti-PD-1 antibody-poorly responsive tumors were defined as tumor volumes larger than 450 mm^3 in 20 mice, and the other 5 mice (N/A; not applicable for evaluation) with tumor too large to be evaluated were excluded from further analysis. Lines inside boxes represent median values of tumor volumes (bars, maximum and minimum tumor volumes). (C) Tumor growth curves of average tumor volumes from the following treatment groups are shown: PBS, *B. longum* 2012, PBS+ anti-PD-1 antibody alone, *B. longum* 420 alone, *B. longum* 420 with anti-PD-1 antibody, after the initial anti-PD-1 treatment. *B. longum* 420 alone significantly suppressed the tumor growth compared to the PBS group at the day 39 (*$p < 0.05$). Each data point presents the average tumor volumes of each group (bars, ±SE). (D) Tumor-infiltrating T cells after treatment in the PD-1 resistance MBT-2 model. The population of tumor-infiltrating CDT, CD8T, Treg cells, KI67-positive T cells, and PD-1-positive T cells was analyzed by flow cytometry (n = 5). Each data point represents the average of the cell frequencies (*$p < 0.05$, bars, ±SE).

to the combination of *B. longum* 420 and anti-PD-1 antibody for tumors poorly responsive to the initial anti-PD-1 antibody treatment. Representative histograms of ICCS are presented in Figure S4.

B440 induced WT1-specific T cell response in cynomolgus monkey

To clinically develop this WT1 oral cancer vaccine, we generated a recombinant *B. longum* 440 displaying a partial human WT1 protein (aa117–419), and produced B440, a pharmaceutical formulation of the lyophilized powder of inactivated *B. longum* 440.[25] We confirmed that oral administration of the expected human equivalent dose of encapsulated B440 induced a WT1-specific T cell response in a cynomolgus monkey (Figure S5). In addition, no obvious adverse effects or signs of toxicity were observed during the experimental period, and B440 was detected by PCR only in feces, and not in blood and urine samples after oral administration (data not shown).

DISCUSSION

Many cancer vaccines have been developed using various platform types such as peptides, protein subunits, DNA, RNA, viral vectors, genetically engineered whole-tumor cells, and antigen-pulsed DCs.[35] These cancer vaccines carry various tumor antigens generally divided into tumor-associated antigens, including cancer-testis antigens (melanoma-associated antigen [MAGE], New York Esophageal Squamous Cell Carcinoma-1 [NY-ESO-1]), oncofetal proteins (carcinoembryonic antigen [CEA], alpha-fetoprotein [AFP]) and normal self-proteins overexpressing in cancer cells (WT1, prostate-specific membrane antigen [PSMA]), and tumor-specific antigens including oncogenic viral antigens (human papilloma virus, Epstein-Barr virus)

and mutated antigens (neoantigens).[36–38] Despite demonstrations of great antitumor activity along with the induction of strong T cell responses in animal studies, the outcomes of numerous clinical trials of those vaccines have been disappointing.[39] Functional inactivation of antitumor T cells is postulated as one of the reasons for failures in the development of cancer vaccines.[40] ICIs, however, show great potential for bringing some previously unsuccessful cancer vaccines back for further clinical development. In the present study, we explored the feasibility of a novel cancer immunotherapy with a WT1 oral cancer vaccine as a combination therapy with following anti-PD-1 antibody and as a monotherapy for anti-PD-1 antibody-poorly responsive tumor and elucidated the immunological mechanism of action of this oral cancer vaccine using a mouse bladder cancer syngeneic tumor model.

First, we confirmed that *B. longum* 420 could induce cellular immunity specific to MBT-2 cells, which naturally express WT1 protein.[26] *B. longum* 420 significantly increased cell proliferation (Figure 1A) and secretion of IFN-γ (Figure 1B) in splenocytes compared to *B. longum* 2012 or PBS. Db126 and mp235 are representative WT1 MHC class I epitopes, and Db126 is known to be restricted to haplotypes of human HLA-A*02:01 and mouse H-2D^b, while mp235 is restricted to human HLA-A*24:02.[30,33] Although mp235 stimulated the proliferation of splenocytes from the vaccinated mice, the stimulation effects of MBT-2 whole cells were higher than the single peptides. MBT-2 whole cells presenting multiple epitopes of WT1 might be able to highly stimulate splenocytes from the *B. longum* 420-vaccinated mice. Also, *B. longum* 420 induced significantly higher *in vitro* cytotoxicity against MBT-2 cells compared to the other mouse groups (Figure 1C).

Next, we investigated the induction of activated CD4$^+$ and CD8$^+$ T cells in spleens and GALT. In spleen, both CD4$^+$ and CD8$^+$ T cells expressing IFN-γ were significantly higher in *B. longum* 420-vaccinated mice compared to the other groups (Figure 1D). In MLNs, both CD4$^+$ and CD8$^+$ T cells expressing IFN-γ in *B. longum* 420-vaccinated mice were significantly higher compared to PBS-treated mice, and, interestingly, CD4$^+$ T cells expressing IFN-γ in *B. longum* 2012, which does not display the heterologous antigen of WT1 protein, were significantly higher than in PBS-treated mice (Figure 1E). As for the DCs, we could not find any significant increase among all groups. However, the most activated DCs in MLN and PP were observed in both *B. longum* 420- and *B. longum* 2012-treated mice. These findings indicated that this vaccine platform using *B. longum* could activate the CD4$^+$ T cell response through the gut immune system. Indeed, Huda et. al.[41] reported that *Bifidobacterium* abundance, especially *B. longum* subspecies *infantis* in early infancy, was positively associated with CD4$^+$ T cell responses to *Bacillus Calmette-Guérin*, tetanus toxoid, and hepatitis B virus vaccines.

B. longum 420 delivers a great length of WT1 protein (aa117–419, Figure 2A), and we previously demonstrated greater antitumor activity of *B. longum* 420 compared to Db126 short peptide vaccine with adjuvant in a mouse prostate cancer syngeneic tumor model.[42] To confirm that *B. longum* 420 could induce CD4$^+$ and CD8$^+$ T cell responses specific to multiple WT1 epitopes, we performed an ICCS assay using eleven WT1 epitope-peptides to identify the specific epitopes (Figures 2A and 2B). The eleven epitopes included known human epitopes (MHC class I epitopes Db126 and mp235 and MHC class II epitope WT1-332) and the top four high-scoring peptides each for H-2K^k (MHC class I of C3H/He mouse) and I-Ak (MHC class II of C3H/He mice) by the SYFPEITHI epitope prediction system (Figure 2B). As a result, the peptides of aa235–243 (mp235), aa273–280, and aa429–437 significantly increased the number of IFN-γ-expressing CD4T and CD8T cells (Figure 2C). Although all three of these epitopes are MHC class I epitopes, both CD4$^+$ and CD8$^+$ T cells were activated. Although the epitope prediction system score did not correlate well with the results of ICCS, nevertheless the ICCS results demonstrated that this oral vaccine could induce CD4$^+$ and CD8$^+$ T cell responses specific to multiple WT1 epitopes. Because of the wide variety of the T cell repertoire and diversity of the MHC haplotype, it is preferable for cancer vaccines to contain tumor antigen composed of multiple epitopes.[43] Furthermore, since the antigen protein delivered by this oral vaccine is taken up inside DCs and processed to multiple epitopes to be presented by DCs, the natural procedure for antigen presentation can induce stronger CTL compared to a short peptide vaccine, which binds directly to MHC protein on the cell surface of professional (DCs) and nonprofessional (T and B cells) antigen-presenting cells.[44]

Our animal experiments successfully confirmed the *in vivo* antitumor activity of the combination of this oral cancer vaccine and following anti-PD-1 antibody. The combination treatment completely suppressed tumor growth in all mice tested (Figures 3B and 3C). In the immunohistochemical study, we observed increased CD4$^+$ and CD8$^+$ TILs in tumor tissues of mice treated with the combination of oral cancer vaccine and anti-PD-1 antibody (Figures 3D and 3E). Also, flow cytometric analysis of TILs revealed that this combination therapy significantly increased CD4$^+$, CD8$^+$, and CD8$^+$CD107a$^+$ TILs (Figure 3F). CD107a is a degranulation marker, and CD8$^+$CD107a$^+$ T cells are considered activated CTLs releasing granzyme and perforin.[34] It is well known that CD4$^+$ helper T cells play a critical role in CTL priming in secondary lymphoid organs.[45] In the present study, we also confirmed that this oral cancer vaccine and *B. longum* itself significantly increased the number of CD4$^+$ T cells in MLN (Figure 1E). Also, in tumor tissues, antigen-specific CD4$^+$ TILs can interact with MHC class II molecule-expressing cells such as myeloid cells and a small subset of tumor cells[46] and modulate the tumor microenvironment (TME) to activate CTL and NK cells. Moreover, CD4$^+$ TILs can directly kill tumor cells through cytolytic mechanisms.[47,48] Indeed, our CD4 and CD8 depletion study revealed that the efficacy of this oral cancer vaccine greatly relies on the function of both CD4$^+$ and CD8$^+$ T cells (Figure 4). On the other hand, CD4$^+$ TILs might differentiate to Treg cells (CD4$^+$CD25$^+$FoxP3$^+$ cells), which suppress antitumor immune responses.[49] Interestingly, this oral cancer vaccine decreased the number of Treg cells in the tumor tissues (Figure 3F). Although the association between *Bifidobacterium and* Treg cells is controversial, some studies indicated that *Bifidobacterium* might suppress Treg cell

activities. For instance, the ratio of $CD8^+$ T cells to Treg cells was increased when $CD8^+$ T cells were activated by *Bifidobacterium*,[50] and *Bifidobacterium* decreased the number of Treg cells in tumor-bearing mice treated with 5-fluorouracil.[51]

There is a great unmet need for clinical treatments for ICI-resistant or -refractory cancers. To address this unmet need and identify the preferred administration sequence of the combination of cancer vaccine and ICI, we performed animal experiments using mice harboring tumors poorly responsive to the initial anti-PD-1 treatment (Figures 5A and 5B). Surprisingly, we found that the oral vaccine alone showed a better outcome compared to the combination of vaccine and anti-PD-1 antibody (Figure 5C). Previously, Kamada et al.[52] reported that PD-1 blockade might amplify Treg cells and cause a hyperprogression disease (HPD) in gastric cancer treated with anti-PD-1 antibody. Consistent with this report, we observed the increase of Treg cells in TILs in continuous anti-PD-1 treatment groups compared to discontinued groups (Figure 5D). These results suggest that monotherapy with cancer vaccine could be preferably selected rather than a combination therapy of cancer vaccine and ICI for ICI-refractory cancers, especially in HPD.

For a better understanding of the clinical feasibility of this oral cancer vaccine, we performed an experiment using cynomolgus monkeys and the encapsulated pharmaceutical formulation B440. The results confirmed that B440 could induce a WT1 protein-specific T cell response in cynomolgus monkeys (Figure S5) without any serious adverse events. These results warrant the further clinical development of this oral cancer vaccine using a *Bifidobacterium* vector alone or as an adjunct with ICIs for patients with advanced urothelial cancer.

MATERIALS AND METHODS

Recombinant *Bifidobacterium*

Genetically modified recombinant *Bifidobacterium*, *B. longum* 420, *B. longum* 440, and *B. longum* 2012, were previously constructed.[19,25] *B. longum* 420 displays a partial murine WT1 protein (aa117–419) by the anchor protein of GL-BP. *B. longum* 440 displays a partial protein of human WT1 protein (aa117–419). *B. longum* 2012 expresses only GL-BP and is used as a control agent.[18] The all three recombinant *Bifidobacterium* bacteria were anaerobically cultured in Gifu anaerobic medium (Nissui, Tokyo, Japan) with 50 μg/mL spectinomycin at 37°C. After the cultivation, these recombinant bacteria were heated for inactivation at 65°C for 5 min.

Cell line

MBT-2, an *N*-4-(5-nitro-2-furyl)-2-thiazolylformamidemurine (FANFT)-induced murine urothelial carcinoma cell line derived from a female C3H/He mouse,[53] was purchased from the Japanese Collection of Research Bioresources (JCRB; Ibaraki, Osaka, Japan) and maintained in Eagle's minimum essential medium supplemented with 10% fetal bovine serum (Sigma-Aldrich Japan, Tokyo, Japan) and 1% penicillin-streptomycin (Nacalai Tesque, Kyoto, Japan). The overexpression of WT1 protein in MBT-2 was previously reported,[26] and we also confirmed that by western blotting analysis. MBT-2 cells naturally express PD-L1 protein, and the expression level was increased by a recombinant protein of interferon-γ (Figure S6).

Oral vaccination

Female C3H/He mice were orally given 100 μL of PBS, 1.0×10^9 CFU of *B. longum* 420, or *B. longum* 2012, 5 times a week for 5 weeks (days 1–5, 8–12, 15–19, 22–26, and 29–33) with a feeding needle. After 2 or 4 weeks of vaccination, mice were euthanized, and MLN and PP were resected to investigate the local gut immune responses. After 5 weeks of vaccination, spleen cells were isolated from other mice to evaluate systemic immune responses with *in vitro* assays.

Proliferation assay

The splenocytes were cultured in RPMI-1640 medium supplemented with 10% FBS, 10 mM HEPES, 100 U/mL penicillin, 100 μg/mL streptomycin, 1 mM nonessential amino acids, 50 μM 2-mercaptoethanol, and 1 mM sodium pyruvate (complete RPMI-1640). To determine the proliferation activity of the splenocytes, we conducted a sodium 2,3-bis (2-methoxy-4-nitro-5-sulfophenyl)-5-[(phenylamino)-carbonyl]-2*H*-tetrazolium) inner salt (XTT) assay. Briefly, 1×10^5 splenocytes were cultured with stimulation with 1×10^4 mitomycin-C-treated MBT-2 cells, Db126 or mp235 peptides (Eurofins Genomics, Tokyo, Japan). After 48 h of cultivation, the cell proliferation was measured by the Cell Proliferation Kit II (XTT) (Roche, Basel, Switzerland). The SI was calculated as the value of stimulated cells divided by the value of non-stimulated cells.

ELISA

Splenocytes were cultivated and stimulated *in vitro*, and the concentrations of IFN-γ in the culture media were measured by a previously described method.[19] Briefly, 4×10^5 splenocyte cells were plated and stimulated with 4×10^4 mitomycin C-treated MBT-2 cells for 3 days. Then the supernatant was collected and frozen at −80°C for the ELISA assay. The secretion of IFN-γ in the supernatant was measured by the Mouse IFN-gamma Quantikine ELISA Kit (R&D Systems, Minneapolis, MN, USA). The procedures were carried out according to the manufacturer's protocol.

ICCS

To obtain single-cell suspensions, the resected tissues of spleen, MLN, and PP were mechanically homogenized and strained. The splenocytes or lymphocytes from MLN (2.0×10^6) were cultured and stimulated with 2.0×10^5 mitomycin-C-treated MBT-2 cells *in vitro*. GolgiStop (BD Biosciences, San Jose, CA, USA) was added to the medium after 26 h of the cell cultivation, and then the cell cultivation was continued for 12 h more. The cells were collected and processed with a BD Cytofix/Cytoperm Plus Fixation/Permeabilization Solution Kit (BD Biosciences) for ICCS assay according to a previously described method.[19] As for the intracellular staining, PE-anti-IFN-γ, PE-anti-TNF-α, and PE-anti-interleukin-2 (IL-2) (BD Biosciences) were used in this study. The stained cells were counted by a Guava flow cytometer (Merck, Darmstadt, Germany).

For epitope peptide screening, splenocytes were isolated after vaccination and re-stimulated with 10 μg/mL of predicted H-2K^k (MHC class I) epitopes or I-Ak (MHC class II) epitopes and known murine and human epitopes *in vitro*. WT1-specific IFN-γ production in spleen T cells was determined by ICCS as described above. Epitope prediction was performed by the SYFPEITHI epitope prediction system (http://www.syfpeithi.de/0-Home.htm). The peptides were purchased from Eurofins Genomics (Figure 2B). As a positive control, the cells were stimulated with concanavalin A (ConA) instead of the peptides.

To investigate the gut immune responses that initiate WT1-specific immunity induced by oral vaccination with recombinant *B. longum* strains, we determined the population and subsets of DCs in MLN and PPs. Single-cell suspensions from MLNs and PPs were blocked with anti-CD16/32 for 20 min. The cells were stained with fluorescein isothiocyanate (FITC)-anti-I-Ak, FITC-anti-CD103, PE-anti-CD11c, APC-anti-CD80, or APC-anti-CD86 antibodies for 30 min in the dark on ice. The staining cells were analyzed by the flow cytometer as described above.

CTL assay

For generating the effector cells, a total of 3.0 × 10^7 splenocytes were cultured with 3.0 × 10^6 mitomycin-C-treated MBT-2 cells with IL-2 for 6 days. The effector cells were co-cultured with MBT-2 cells as the target cells at a ratio of 5:1, 10:1, and 20:1 for 8 h. After the cell cultivation, the supernatant was collected for measuring the CTL activity with a lactate dehydrogenase (LDH) cytotoxicity assay kit (CytoTox 96 Non-Radioactive Cytotoxicity Assay; Promega, Fitchburg, WI, USA) according to the manufacturer's protocol. The percentage of specific cell lysis was calculated as experimental release − effector spontaneous release − target spontaneous release/target maximum release − target spontaneous release × 100.

Animal experiment for combination therapy

To explore the *in vivo* antitumor activity of the combination of *B. longum* 420 and anti-PD-1 antibody against urothelial cancer, we employed an MBT-2 mouse bladder cancer syngeneic subcutaneous tumor model. One million MBT-2 cells were subcutaneously injected into female C3H/He mice at day 0. A total of 35 mice with MBT-2 subcutaneous tumor were randomly assigned to 5 treatment groups (n = 7): *B. longum* 420+anti-PD-1, *B. longum* 420, *B. longum* 2012, PBS+anti-PD-1, and PBS at day 7, and then oral administrations were carried out as described above (Figure 3A). Anti-mouse PD-1 antibody (*InVivo*Plus anti-mouse PD-1, clone RMP1-14, Bio X Cell, West Lebanon, NH, USA) was used for the anti-PD-1 treatment, and rat IgG2al (*InVivo*Plus Rat IgG2a Isotype Control, clone 2A3, Bio X Cell) was used as an isotype control. Two hundred milligrams of anti-PD-1 antibody or IgG isotype control was intraperitoneally injected into mice at days 11, 14, 17, 21, and 24. Tumor volume was measured by the calculation formula of (longest diameter) × (shortest diameter)^2 × 0.5. Mice were euthanized when their tumors grew larger than 20-mm diameter, and Kaplan-Meier survival curves were generated.

CD4$^+$ and CD8$^+$ T cell depletion study

Another set of mice were intraperitoneally injected with anti-CD4, anti-CD8, or IgG isotype control at days −1, 1, 7, and 11 (n = 5, respectively) as described elsewhere. Both antibodies were purchased from Bio X Cell. At day 0, 1 × 10^6 MBT-2 was injected into the mice, and oral vaccination with 1.0 × 10^9 of recombinant *B. longum* 420 was carried out in the following weeks. Figure 4A shows the experimental design.

Immunohistochemical study

Another set of mice was injected with 2 × 10^6 MBT-2 and treated by the same method described above. Tumors were resected and divided into two pieces, and half was fixed with 4% paraformaldehyde-PBS and embedded in paraffin. Another piece of tumor was used for flow cytometry as described below. Immunohistochemical staining was performed as in our previous study.[25] Anti-mouse CD4 antibody (1:1,000, Abcam, Cambridge, UK) and anti-mouse CD8a antibody (1:400, Cell Signaling Technology Japan, Tokyo, Japan) were used in the immunohistochemical staining. The tissue slides were observed with a BZ-X710 microscope (Keyence, Osaka, Japan).

Tumor-infiltrating lymphocytes

To obtain the single-cell suspensions from tumors, resected tumor tissues were mechanically homogenized with incellPREP (incellDx, San Carlos, CA, USA) and strained. The single-cell suspensions in 10 mM EDTA-PBS were blocked with anti-CD16/32 for 20 min and stained with PerCP-anti-CD3, FITC-anti-CD4, APC-anti-CD8, and PE-anti-CD107a antibodies for 30 min. The stained cells were counted by flow cytometry as described above. For Treg cell staining, the cells were stained with PerCP-anti-CD3, FITC-anti-CD4, and APC-anti-CD25 antibodies. For intracellular staining, the cells were permeabilized with BD Fixation/Permeabilization Solution and stained with PE-anti-FoxP3 (BD Biosciences). The stained cells were counted by flow cytometer.

Tumor model poorly responsive to anti-PD-1 antibody

To investigate the therapeutic efficacy of *B. longum* 420 in tumor poorly responsive to anti-PD-1 antibody, a total of 60 mice were inoculated with 1 × 106 MBT-2 cells and received anti-PD-1 therapy ahead of oral vaccination with *B. longum* 420. Twenty days after tumor inoculation, 20 out of 60 mice were selected as harboring anti-PD-1 antibody-poorly responsive tumors based on tumor size (>450 mm^3, Figures 5A and 5B) and then randomly assigned to 4 treatment groups: PBS, *B. longum* 420 alone, anti-PD-1 alone, and combination of *B. longum* 420 and anti-PD-1 antibody (n = 5, respectively). The other 40 mice harboring small tumor volume (<450 mm^3, as anti-PD-1 antibody responsive) or too large volume (N/A [not applicable for evaluation]) to be evaluated were excluded from the study (Figures 5A and 5B). Figure 5B shows the dot plot graph of the individual tumor volumes of the 60 mice at day 20 (anti-PD-1 antibody responsive: 35 mice, anti-PD-1 antibody poorly responsive: 20 mice, N/A: 5 mice). Then, 40 days after tumor inoculation, tumors were resected and homogenized with a gentleMACS Tissue Dissociator (Miltenyi Biotec, Bergisch Gladbach,

Germany) according to the manufacturer's manual. TILs were measured by flow cytometry.

Preclinical study using cynomolgus monkeys

A pharmaceutical formulation of B440, as a lyophilized powder of inactivated *B. longum* 440, a recombinant strain that expresses modified human WT1 protein, was used for our preclinical study.[25] Capsules containing B440 were orally administered at low dose (39 mg/kg/day) or high dose (77 mg/kg/day), equivalent to 1.0 g or 2.0 g for human (60 kg/body/day) doses, to cynomolgus monkeys (n = 1, respectively) 5 times a week for 4 weeks. After the final oral administration, peripheral blood was collected, and peripheral blood mononuclear cells (PBMCs) were isolated by gradient centrifugation. One million monkey PBMCs were seeded into 96-well plates in complete RPMI-1640 medium with 3 μg/mL human WT1 protein (OriGene, Rockville, MD, USA) and 1 μg/mL CD28/49d (BD Biosciences) for 12 h. After culture, cells were washed with Fcg receptor inhibitor (Thermo Fisher Scientific, Waltham, MA, USA) and stained with PerCP-anti-human CD3, FITC-anti-human CD4, and APC-anti-human CD8 (BD Biosciences, respectively) in staining buffer. After staining, cells were fixed and stained with PE-anti-human IFN-γ (BD Biosciences) and analyzed by flow cytometry.

Statistical analysis

One-way ANOVA followed by the Tukey-Kramer method was employed for the comparisons between multiple groups. The log-rank test on Kaplan-Meier curves was employed for the statistical analysis of the survival between groups. Differences among experimental groups were considered significant when $p < 0.05$.

Guidelines and regulations

All experiments and methods were performed in accordance with the relevant guidelines and regulations, and all experimental protocols, including animal experimental designs and procedures, were reviewed and approved by the institutional ethics and animal welfare committees of the Kobe University Graduate School of Medicine.

SUPPLEMENTAL INFORMATION

Supplemental information can be found online at https://doi.org/10.1016/j.omto.2021.08.009.

ACKNOWLEDGMENTS

This research was supported by the Translational Research Program, Strategic Promotion for Practical Application of Innovative Medical Technology (TR-SPRINT) from the Japan Agency for Medical Research and Development (AMED) under grant numbers 17lm0203036, 19lm0203091h0001, and 20lm0203091h0002 (to T.S., Y.H., T.K., and M.F.).

AUTHOR CONTRIBUTIONS

T.S. conceptualized the study. K.K., Y.H., T.K., and T.S. developed the methodology. K.K.. M.T.. S.K., and H.D. carried out the experiments. T.S. and K.K conducted the experiments and wrote the paper. T.S. and M.F. supervised the study.

DECLARATION OF INTERESTS

The authors declare no competing interests.

REFERENCES

1. Goldin, B.R., and Gorbach, S.L. (1980). Effect of Lactobacillus acidophilus dietary supplements on 1,2-dimethylhydrazine dihydrochloride-induced intestinal cancer in rats. J. Natl. Cancer Inst. *64*, 263–265.
2. Cheng, W.Y., Wu, C.Y., and Yu, J. (2020). The role of gut microbiota in cancer treatment: friend or foe? Gut *69*, 1867–1876.
3. Fong, W., Li, Q., and Yu, J. (2020). Gut microbiota modulation: a novel strategy for prevention and treatment of colorectal cancer. Oncogene *39*, 4925–4943.
4. Aarnoutse, R., Ziemons, J., Penders, J., Rensen, S.S., de Vos-Geelen, J., and Smidt, M.L. (2019). The Clinical Link between Human Intestinal Microbiota and Systemic Cancer Therapy. Int. J. Mol. Sci. *20*, E4145.
5. Wang, J., Yang, H.R., Wang, D.J., and Wang, X.X. (2020). Association between the gut microbiota and patient responses to cancer immune checkpoint inhibitors. Oncol. Lett. *20*, 342.
6. Peng, Z., Cheng, S., Kou, Y., Wang, Z., Jin, R., Hu, H., Zhang, X., Gong, J.F., Li, J., Lu, M., et al. (2020). The Gut Microbiome Is Associated with Clinical Response to Anti-PD-1/PD-L1 Immunotherapy in Gastrointestinal Cancer. Cancer Immunol. Res. *8*, 1251–1261.
7. Sivan, A., Corrales, L., Hubert, N., Williams, J.B., Aquino-Michaels, K., Earley, Z.M., Benyamin, F.W., Lei, Y.M., Jabri, B., Alegre, M.L., et al. (2015). Commensal Bifidobacterium promotes antitumor immunity and facilitates anti-PD-L1 efficacy. Science *350*, 1084–1089.
8. Roy, S., and Trinchieri, G. (2017). Microbiota: a key orchestrator of cancer therapy. Nat. Rev. Cancer *17*, 271–285.
9. Mukaida, N. (2014). Intestinal microbiota: unexpected alliance with tumor therapy. Immunotherapy 6, 231–233.
10. Daillère, R., Vétizou, M., Waldschmitt, N., Yamazaki, T., Isnard, C., Poirier-Colame, V., Duong, C.P.M., Flament, C., Lepage, P., Roberti, M.P., et al. (2016). Enterococcus hirae and Barnesiella intestinihominis Facilitate Cyclophosphamide-Induced Therapeutic Immunomodulatory Effects. Immunity *45*, 931–943.
11. Paulos, C.M., Wrzesinski, C., Kaiser, A., Hinrichs, C.S., Chieppa, M., Cassard, L., Palmer, D.C., Boni, A., Muranski, P., Yu, Z., et al. (2007). Microbial translocation augments the function of adoptively transferred self/tumor-specific CD8+ T cells via TLR4 signaling. J. Clin. Invest. *117*, 2197–2204.
12. Vétizou, M., Pitt, J.M., Daillère, R., Lepage, P., Waldschmitt, N., Flament, C., Rusakiewicz, S., Routy, B., Roberti, M.P., Duong, C.P., et al. (2015). Anticancer immunotherapy by CTLA-4 blockade relies on the gut microbiota. Science *350*, 1079–1084.
13. Bumann, D., Behre, C., Behre, K., Herz, S., Gewecke, B., Gessner, J.E., von Specht, B.U., and Baumann, U. (2010). Systemic, nasal and oral live vaccines against Pseudomonas aeruginosa: a clinical trial of immunogenicity in lower airways of human volunteers. Vaccine *28*, 707–713.
14. Seo, S.U., Kim, J.J., Yang, H., Kwon, H.J., Yang, J.Y., Curtiss Iii, R., and Kweon, M.N. (2012). Effective protection against secondary pneumococcal pneumonia by oral vaccination with attenuated Salmonella delivering PspA antigen in mice. Vaccine *30*, 6816–6823.
15. Dharmasena, M.N., Osorio, M., Filipova, S., Marsh, C., Stibitz, S., and Kopecko, D.J. (2016). Stable expression of Shigella dysenteriae serotype 1 O-antigen genes integrated into the chromosome of live Salmonella oral vaccine vector Ty21a. Pathog. Dis. *74*, ftw098.
16. Ding, C., Ma, J., Dong, Q., and Liu, Q. (2018). Live bacterial vaccine vector and delivery strategies of heterologous antigen: A review. Immunol. Lett. *197*, 70–77.
17. Yamamoto, S., Wada, J., Katayama, T., Jikimoto, T., Nakamura, M., Kinoshita, S., Lee, K.M., Kawabata, M., and Shirakawa, T. (2010). Genetically modified Bifidobacterium displaying Salmonella-antigen protects mice from lethal challenge of Salmonella Typhimurium in a murine typhoid fever model. Vaccine *28*, 6684–6691.
18. Takei, S., Omoto, C., Kitagawa, K., Morishita, N., Katayama, T., Shigemura, K., Fujisawa, M., Kawabata, M., Hotta, H., and Shirakawa, T. (2014). Oral administration of genetically modified Bifidobacterium displaying HCV-NS3 multi-epitope fusion

protein could induce an HCV-NS3-specific systemic immune response in mice. Vaccine *32*, 3066–3074.

19. Kitagawa, K., Oda, T., Saito, H., Araki, A., Gonoi, R., Shigemura, K., Hashii, Y., Katayama, T., Fujisawa, M., and Shirakawa, T. (2017). Development of oral cancer vaccine using recombinant Bifidobacterium displaying Wilms' tumor 1 protein. Cancer Immunol. Immunother. *66*, 787–798.

20. Yang, L., Han, Y., Suarez Saiz, F., and Minden, M.D. (2007). A tumor suppressor and oncogene: the WT1 story. Leukemia *21*, 868–876.

21. Inoue, K., Sugiyama, H., Ogawa, H., Nakagawa, M., Yamagami, T., Miwa, H., Kita, K., Hiraoka, A., Masaoka, T., Nasu, K., et al. (1994). WT1 as a new prognostic factor and a new marker for the detection of minimal residual disease in acute leukemia. Blood *84*, 3071–3079.

22. Cheever, M.A., Allison, J.P., Ferris, A.S., Finn, O.J., Hastings, B.M., Hecht, T.T., Mellman, I., Prindiville, S.A., Viner, J.L., Weiner, L.M., and Matrisian, L.M. (2009). The prioritization of cancer antigens: a national cancer institute pilot project for the acceleration of translational research. Clin. Cancer Res. *15*, 5323–5337.

23. Hiramatsu, Y., Hosono, A., Konno, T., Nakanishi, Y., Muto, M., Suyama, A., Hachimura, S., Sato, R., Takahashi, K., and Kaminogawa, S. (2011). Orally administered Bifidobacterium triggers immune responses following capture by CD11c(+) cells in Peyer's patches and cecal patches. Cytotechnology *63*, 307–317.

24. Matson, V., Fessler, J., Bao, R., Chongsuwat, T., Zha, Y.Y., Alegre, M.L., Luke, J.J., and Gajewski, T.F. (2018). The commensal microbiome is associated with anti-PD-1 efficacy in metastatic melanoma patients. Science *359*, 104–108.

25. Kitagawa, K., Gonoi, R., Tatsumi, M., Kadowaki, M., Katayama, T., Hashii, Y., Fujisawa, M., and Shirakawa, T. (2019). Preclinical Development of a WT1 Oral Cancer Vaccine Using a Bacterial Vector to Treat Castration-Resistant Prostate Cancer. Mol. Cancer Ther. *18*, 980–990.

26. Yu, D.S., and Chang, S.Y. (2002). The expression of oncoproteins in transitional cell carcinoma: its correlation with pathological behavior, cell cycle and drug resistance. Urol. Int. *69*, 46–50.

27. Gettinger, S.N., Horn, L., Gandhi, L., Spigel, D.R., Antonia, S.J., Rizvi, N.A., Powderly, J.D., Heist, R.S., Carvajal, R.D., Jackman, D.M., et al. (2015). Overall Survival and Long-Term Safety of Nivolumab (Anti-Programmed Death 1 Antibody, BMS-936558, ONO-4538) in Patients With Previously Treated Advanced Non-Small-Cell Lung Cancer. J. Clin. Oncol. *33*, 2004–2012.

28. Muro, K., Chung, H.C., Shankaran, V., Geva, R., Catenacci, D., Gupta, S., Eder, J.P., Golan, T., Le, D.T., Burtness, B., et al. (2016). Pembrolizumab for patients with PD-L1-positive advanced gastric cancer (KEYNOTE-012): a multicentre, open-label, phase 1b trial. Lancet Oncol. *17*, 717–726.

29. Fradet, Y., Bellmunt, J., Vaughn, D.J., Lee, J.L., Fong, L., Vogelzang, N.J., Climent, M.A., Petrylak, D.P., Choueiri, T.K., Necchi, A., et al. (2019). Randomized phase III KEYNOTE-045 trial of pembrolizumab versus paclitaxel, docetaxel, or vinflunine in recurrent advanced urothelial cancer: results of >2 years of follow-up. Ann. Oncol. *30*, 970–976.

30. Oka, Y., Udaka, K., Tsuboi, A., Elisseeva, O.A., Ogawa, H., Aozasa, K., Kishimoto, T., and Sugiyama, H. (2000). Cancer immunotherapy targeting Wilms' tumor gene WT1 product. J. Immunol. *164*, 1873–1880.

31. Dredge, K., Marriott, J.B., Todryk, S.M., Muller, G.W., Chen, R., Stirling, D.I., and Dalgleish, A.G. (2002). Protective antitumor immunity induced by a costimulatory thalidomide analog in conjunction with whole tumor cell vaccination is mediated by increased Th1-type immunity. J. Immunol. *168*, 4914–4919.

32. Ruiz, L., Delgado, S., Ruas-Madiedo, P., Sánchez, B., and Margolles, A. (2017). Bifidobacteria and Their Molecular Communication with the Immune System. Front. Microbiol. *8*, 2345.

33. Fujiki, F., Oka, Y., Tsuboi, A., Kawakami, M., Kawakatsu, M., Nakajima, H., Elisseeva, O.A., Harada, Y., Ito, K., Li, Z., et al. (2007). Identification and characterization of a WT1 (Wilms Tumor Gene) protein-derived HLA-DRB1*0405-restricted 16-mer helper peptide that promotes the induction and activation of WT1-specific cytotoxic T lymphocytes. J. Immunother. *30*, 282–293.

34. Alter, G., Malenfant, J.M., and Altfeld, M. (2004). CD107a as a functional marker for the identification of natural killer cell activity. J. Immunol. Methods *294*, 15–22.

35. Melief, C.J., van Hall, T., Arens, R., Ossendorp, F., and van der Burg, S.H. (2015). Therapeutic cancer vaccines. J. Clin. Invest. *125*, 3401–3412.

36. Krishnadas, D.K., Shusterman, S., Bai, F., Diller, L., Sullivan, J.E., Cheerva, A.C., George, R.E., and Lucas, K.G. (2015). A phase I trial combining decitabine/dendritic cell vaccine targeting MAGE-A1, MAGE-A3 and NY-ESO-1 for children with relapsed or therapy-refractory neuroblastoma and sarcoma. Cancer Immunol. Immunother. *64*, 1251–1260.

37. Turriziani, M., Fantini, M., Benvenuto, M., Izzi, V., Masuelli, L., Sacchetti, P., Modesti, A., and Bei, R. (2012). Carcinoembryonic antigen (CEA)-based cancer vaccines: recent patents and antitumor effects from experimental models to clinical trials. Recent Patents Anticancer Drug Discov. *7*, 265–296.

38. Aggarwal, C., Cohen, R.B., Morrow, M.P., Kraynyak, K.A., Sylvester, A.J., Knoblock, D.M., Bauml, J.M., Weinstein, G.S., Lin, A., Boyer, J., et al. (2019). Immunotherapy Targeting HPV16/18 Generates Potent Immune Responses in HPV-Associated Head and Neck Cancer. Clin. Cancer Res. *25*, 110–124.

39. Rosenberg, S.A., Yang, J.C., and Restifo, N.P. (2004). Cancer immunotherapy: moving beyond current vaccines. Nat. Med. *10*, 909–915.

40. Melero, I., Shuford, W.W., Newby, S.A., Aruffo, A., Ledbetter, J.A., Hellström, K.E., Mittler, R.S., and Chen, L. (1997). Monoclonal antibodies against the 4-1BB T-cell activation molecule eradicate established tumors. Nat. Med. *3*, 682–685.

41. Huda, M.N., Ahmad, S.M., Alam, M.J., Khanam, A., Kalanetra, K.M., Taft, D.H., Raqib, R., Underwood, M.A., Mills, D.A., and Stephensen, C.B. (2019). Bifidobacterium Abundance in Early Infancy and Vaccine Response at 2 Years of Age. Pediatrics *143*, e20181489.

42. Shirakawa, T., and Kitagawa, K. (2018). Antitumor effect of oral cancer vaccine with Bifidobacterium delivering WT1 protein to gut immune system is superior to WT1 peptide vaccine. Hum. Vaccin. Immunother. *14*, 159–162.

43. Noguchi, M., Arai, G., Matsumoto, K., Naito, S., Moriya, F., Suekane, S., Komatsu, N., Matsueda, S., Sasada, T., Yamada, A., et al. (2015). Phase I trial of a cancer vaccine consisting of 20 mixed peptides in patients with castration-resistant prostate cancer: dose-related immune boosting and suppression. Cancer Immunol. Immunother. *64*, 493–505.

44. Melief, C.J., and van der Burg, S.H. (2008). Immunotherapy of established (pre)malignant disease by synthetic long peptide vaccines. Nat. Rev. Cancer *8*, 351–360.

45. Yu, P., Spiotto, M.T., Lee, Y., Schreiber, H., and Fu, Y.X. (2003). Complementary role of CD4+ T cells and secondary lymphoid tissues for cross-presentation of tumor antigen to CD8+ T cells. J. Exp. Med. *197*, 985–995.

46. Borst, J., Ahrends, T., Bąbała, N., Melief, C.J.M., and Kastenmüller, W. (2018). $CD4^+$ T cell help in cancer immunology and immunotherapy. Nat. Rev. Immunol. *18*, 635–647.

47. Melssen, M., and Slingluff, C.L., Jr. (2017). Vaccines targeting helper T cells for cancer immunotherapy. Curr. Opin. Immunol. *47*, 85–92.

48. Kennedy, R., and Celis, E. (2008). Multiple roles for CD4+ T cells in anti-tumor immune responses. Immunol. Rev. *222*, 129–144.

49. Clarke, S.L., Betts, G.J., Plant, A., Wright, K.L., El-Shanawany, T.M., Harrop, R., Torkington, J., Rees, B.I., Williams, G.T., Gallimore, A.M., and Godkin, A.J. (2006). CD4+CD25+FOXP3+ regulatory T cells suppress anti-tumor immune responses in patients with colorectal cancer. PLoS ONE *1*, e129.

50. Rong, Y., Dong, Z., Hong, Z., Jin, Y., Zhang, W., Zhang, B., Mao, W., Kong, H., Wang, C., Yang, B., et al. (2017). Reactivity toward Bifidobacterium longum and Enterococcus hirae demonstrate robust $CD8^+$ T cell response and better prognosis in HBV-related hepatocellular carcinoma. Exp. Cell Res. *358*, 352–359.

51. Xie, N., Wang, Y., Wang, Q., Li, F.R., and Guo, B. (2012). Lipoteichoic acid of Bifidobacterium in combination with 5-fluorouracil inhibit tumor growth and relieve the immunosuppression. Bull. Cancer *99*, E55–E63.

52. Kamada, T., Togashi, Y., Tay, C., Ha, D., Sasaki, A., Nakamura, Y., Sato, E., Fukuoka, S., Tada, Y., Tanaka, A., et al. (2019). $PD-1^+$ regulatory T cells amplified by PD-1 blockade promote hyperprogression of cancer. Proc. Natl. Acad. Sci. USA *116*, 9999–10008.

53. Soloway, M.S. (1977). Intravesical and systemic chemotherapy of murine bladder cancer. Cancer Res. *37*, 2918–2929.

OMTO, Volume *22*

Supplemental information

An oral cancer vaccine using a *Bifidobacterium* vector suppresses tumor growth in a syngeneic mouse bladder cancer model

Koichi Kitagawa, Maho Tatsumi, Mako Kato, Shota Komai, Hazuki Doi, Yoshiko Hashii, Takane Katayama, Masato Fujisawa, and Toshiro Shirakawa

Supplemental Information

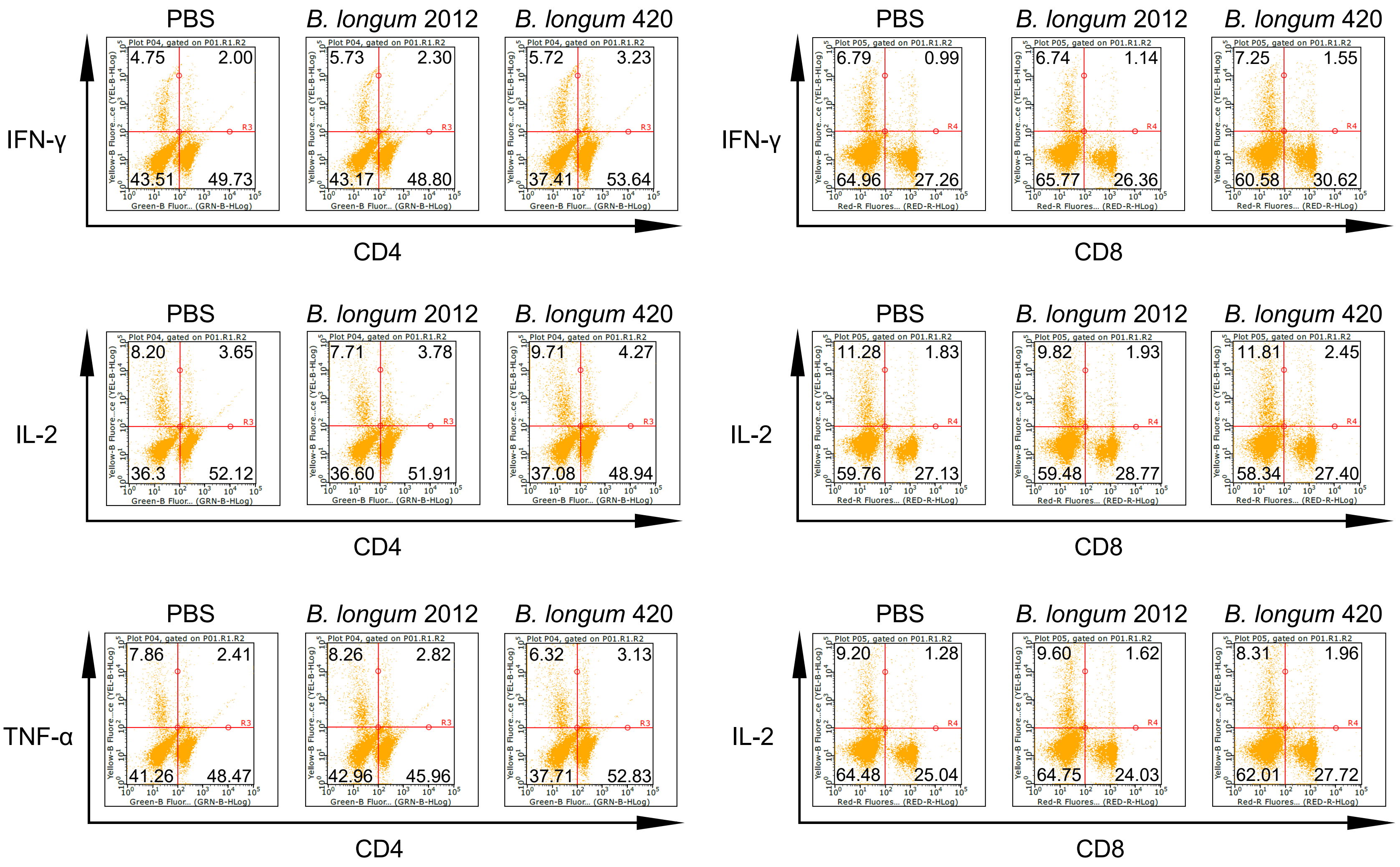

Figure S1. Representative histograms of ICCS for splenocytes

Representative dot plots and gating of intracellular cytokine staining (ICCS) for T cells in splenocytes.

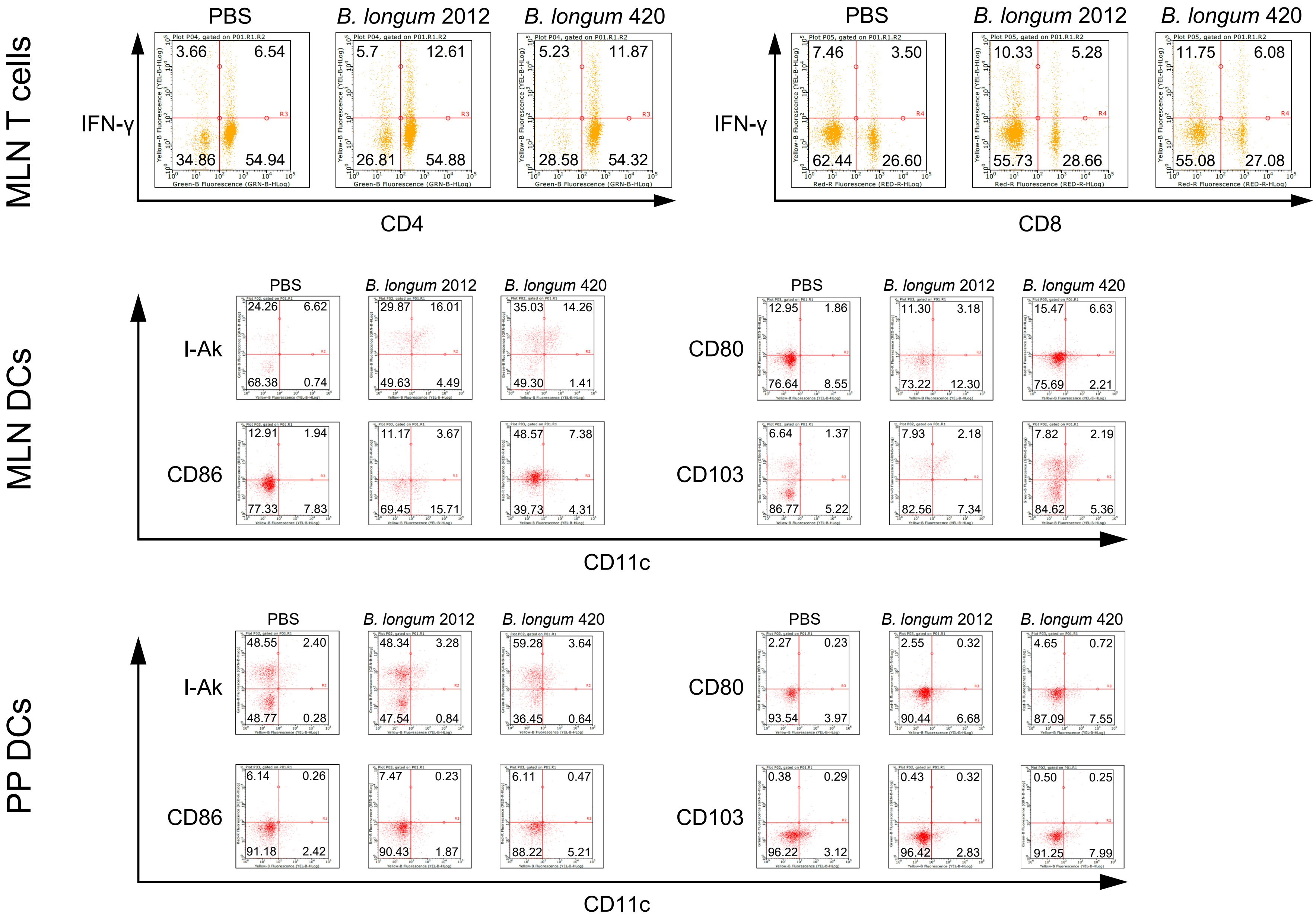

Figure S2. Representative histograms of ICCS for T cells DCs in MLNs and PPs
Representative dot plots and gating for intracellular cytokine staining (ICCS) for T cells in mesenteric lymph nodes (MLNs) and Peyer's patches (PPs), and dendritic cells (DCs) in MLNs and PPs.

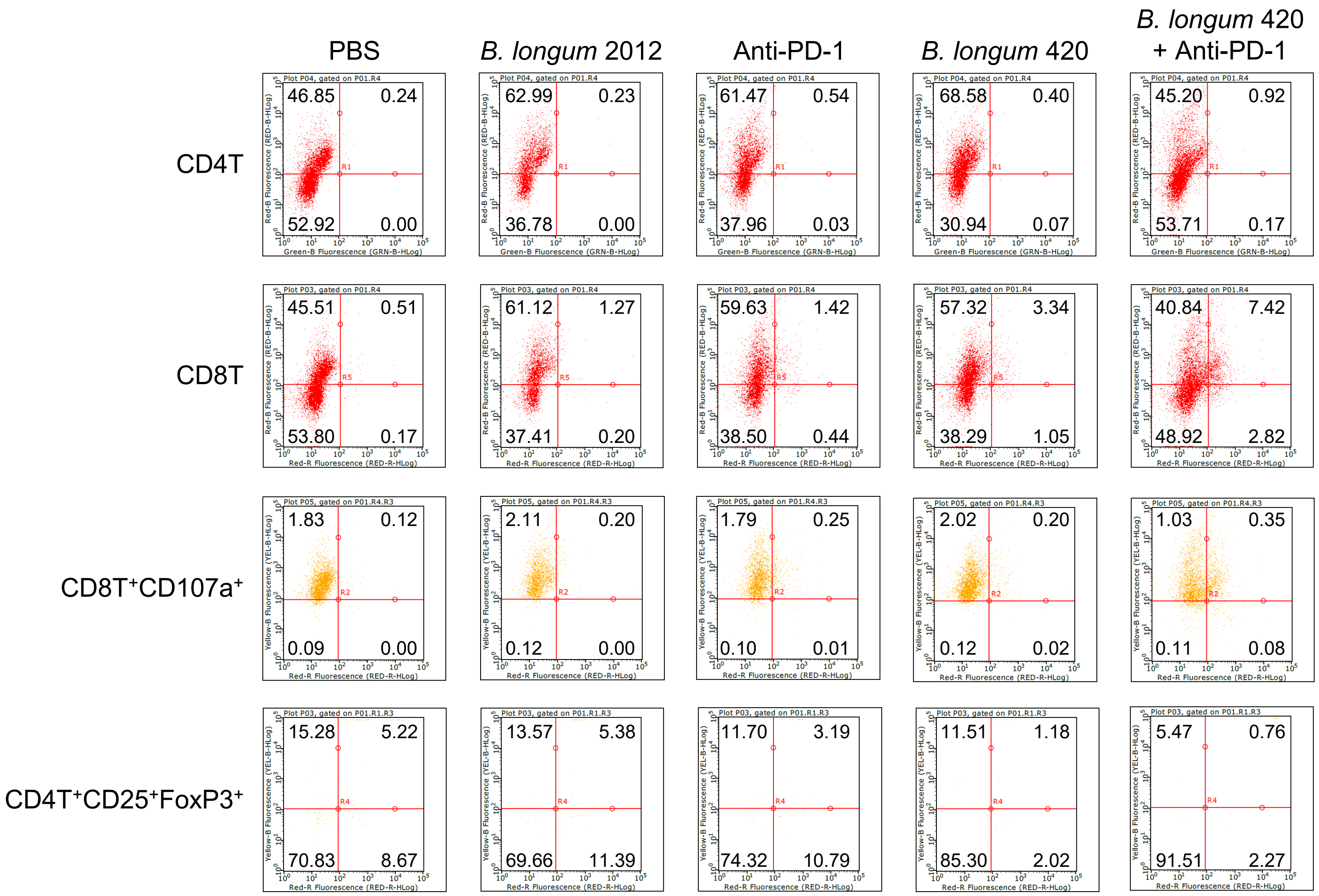

Figure S3. Representative histograms of FMC analysis for TIL in MBT-2 tumor tissues
Representative dot plots and gating of flow cytometric (FCM) analysis for tumor infiltrating lymphocytes (TILs) in MBT-2 tumors after combination therapy with *B. longum* 420 and anti-PD-1 antibody.

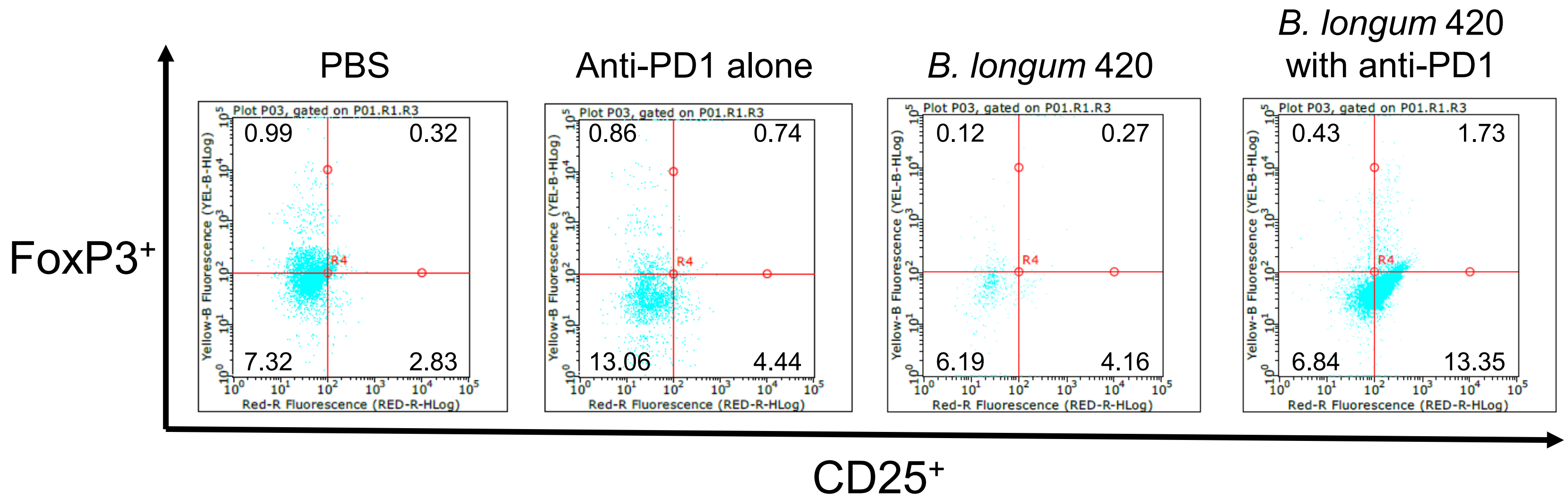

Figure S4. Representative histograms of FMC for TIL in MBT-2 tumor tissues
Representative dot plots and gating of flow cytometric (FCM) analysis for tumor infiltrating regulatory T cells in MBT-2 tumors poorly responsive to anti-PD-1 antibody.

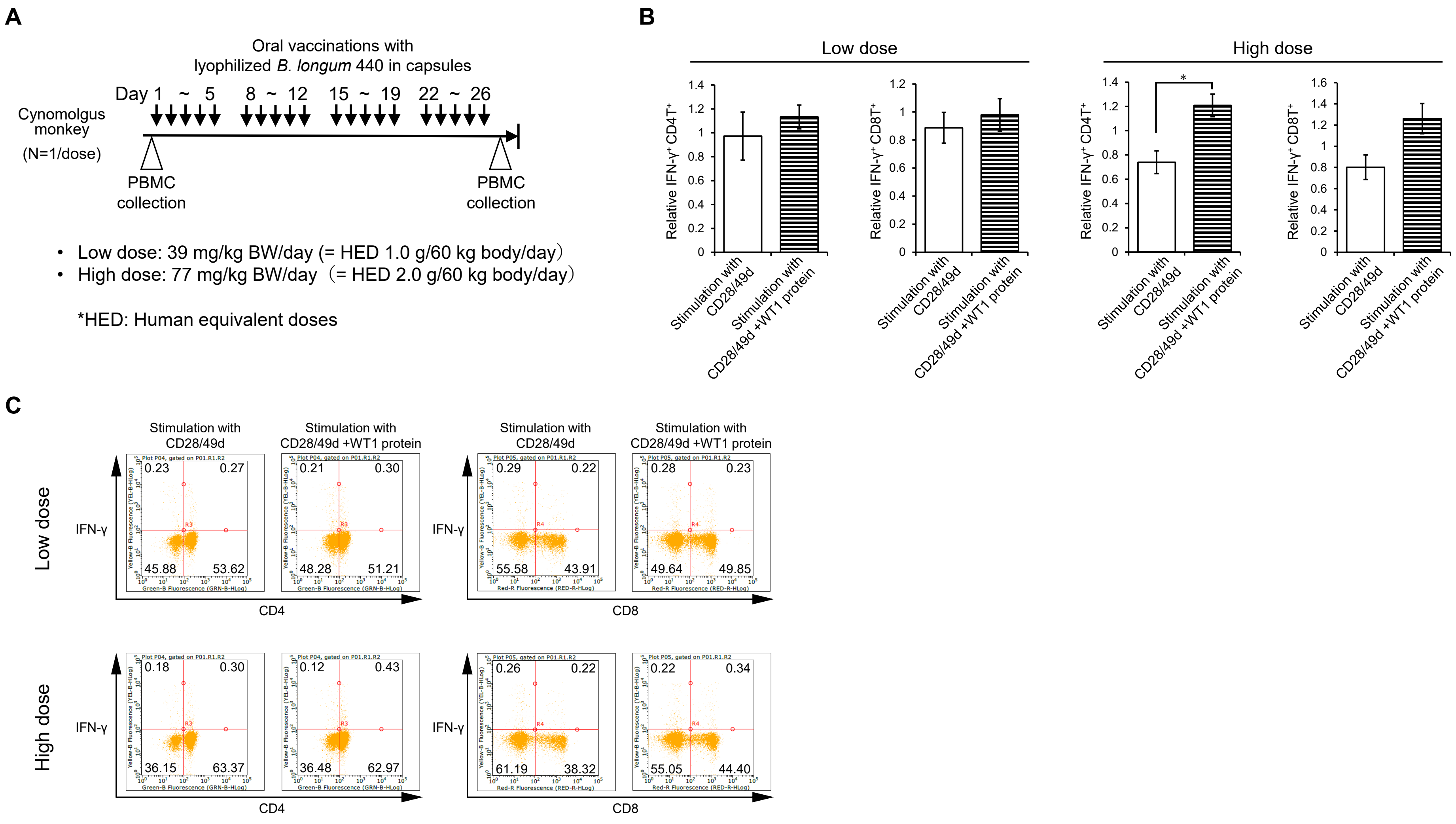

Figure S5. B440 induced WT1-specific T cell response in cynomolgus monkey

The B440, a lyophilized *B. longum* 440 in capsules was orally administrated into cynomolgus monkeys, at a low dose (39 mg/kg/day) and high dose (77 mg/kg/day), which is equivalent to 1.0 g or 2.0 g for human (60 kg/body/day) doses, five times a week for four weeks (A). After final oral administration, peripheral blood mononuclear cells (PBMCs) were isolated for ICCS assay. As a result, the number of $CD4^+IFN\text{-}\gamma^+$ T cells was significantly increased after stimulation with WT1 protein in the cynomolgus monkey vaccinated with a high dose of B440 (B). Also, the number of $CD8^+IFN\text{-}\gamma^+$ T cells was increased after stimulation with WT1 protein in the cynomolgus monkey vaccinated with high dose B440, but this was not statistically significant (B). (C) Representative histograms and gating for intracellular cytokine staining (ICCS) of monkey PBMCs after oral administration of *B. longum* 440.

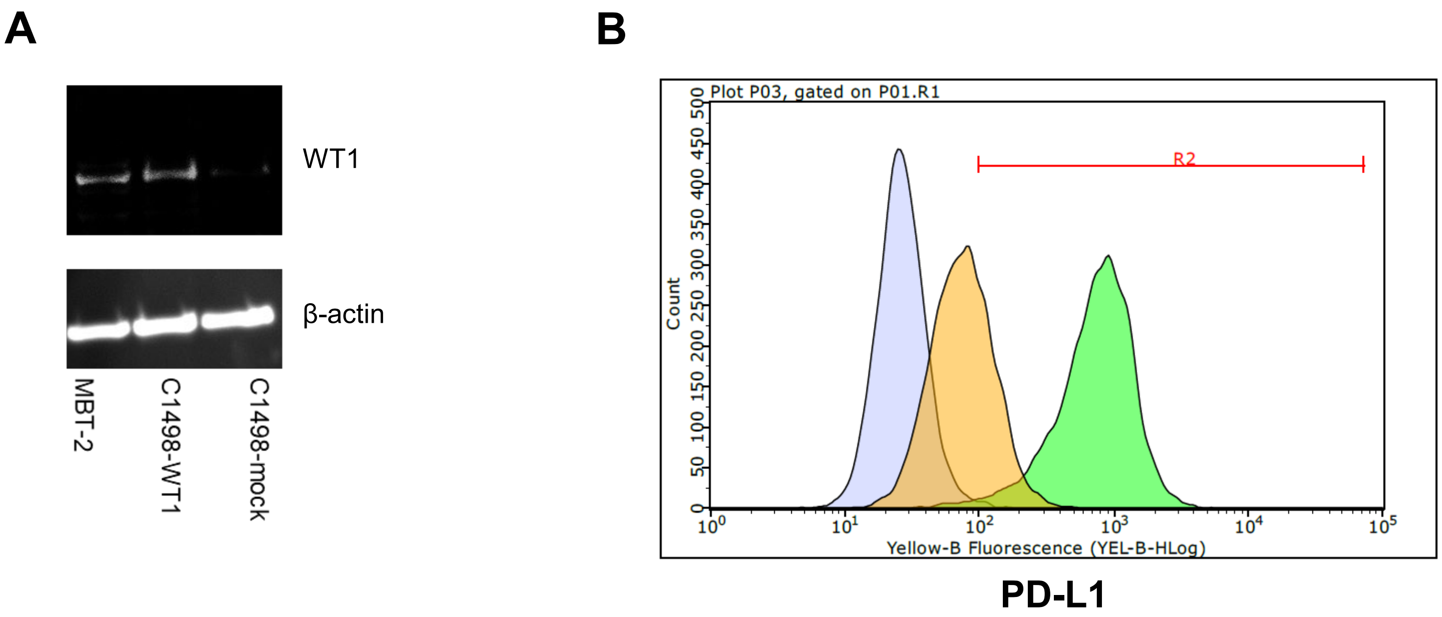

Figure S6. *In vitro* expressions of WT1 and PD-L1 proteins in MBT-2 cells

A) Expression of WT1 protein in MBT-2. The expression of WT1 protein was determined by western blotting. WT1 protein was detected at 55 kDa in MBT-2 and C1498-WT1 but not in C1498-mock.

B) PD-L1 expression on MBT-2 cells. MBT-2 cells were treated with/without IFN-γ and stained with anti-mouse PD-L1 antibody in vitro. Green: MBT-2 cells stained with PD-L1 after culture with 20 ng/ml IFN-γ. Orange: MBT-2 cells stained with PD-L1 after culture with 0 ng/ml IFN-γ Light blue: MBT-2 cells stained with isotype control IgG after culture with 20 ng/ml IFN-γ.
